# Dissecting the Effect of a 3D Microscaffold on the Transcriptome of Neural Stem Cells with Computational Approaches: A Focus on Mechanotransduction

**DOI:** 10.3390/ijms21186775

**Published:** 2020-09-15

**Authors:** Federica Rey, Cecilia Pandini, Bianca Barzaghini, Letizia Messa, Toniella Giallongo, Orietta Pansarasa, Stella Gagliardi, Matteo Brilli, Gian Vincenzo Zuccotti, Cristina Cereda, Manuela Teresa Raimondi, Stephana Carelli

**Affiliations:** 1 Department of Biomedical and Clinical Sciences “L. Sacco”, University of Milan, Via Grassi 74, 20157 Milan, Italy; federica.rey@unimi.it (F.R.); toniella.giallongo@unimi.it (T.G.); gianvincenzo.zuccotti@unimi.it (G.V.Z.); 2 Pediatric Research Center “Romeo ed Enrica Invernizzi”, University of Milano, Via G.B. Grassi 74, 20157 Milano, Italy; matteo.brilli@unimi.it; 3 Genomic and Post-Genomic Center, IRCCS Mondino Foundation, 27100 Pavia, Italy; cecilia.pandini@mondino.it (C.P.); orietta.pansarasa@mondino.it (O.P.); Stella.gagliardi@mondino.it (S.G.); 4 Department of Biology and Biotechnology “L. Spallanzani”, University of Pavia, 27100 Pavia, Italy; 5 Department of Chemistry, Materials and Chemical Engineering “Giulio Natta”, Politecnico di Milano, 20133 Milano, Italy; bianca.barzaghini@polimi.it (B.B.); letizia.messa@mail.polimi.it (L.M.); manuela.raimondi@polimi.it (M.T.R.); 6 Department of Biosciences, University of Milan, Via Celoria 26, 20133 Milano, Italy

**Keywords:** mechanotransduction, scaffolds, neural stem cells, RNA-Seq, computational genomics, signal transduction, niche, mechanobiology, regenerative medicine

## Abstract

3D cell cultures are becoming more and more important in the field of regenerative medicine due to their ability to mimic the cellular physiological microenvironment. Among the different types of 3D scaffolds, we focus on the Nichoid, a miniaturized scaffold with a structure inspired by the natural staminal niche. The Nichoid can activate cellular responses simply by subjecting the cells to mechanical stimuli. This kind of influence results in different cellular morphology and organization, but the molecular bases of these changes remain largely unknown. Through RNA-Seq approach on murine neural precursors stem cells expanded inside the Nichoid, we investigated the deregulated genes and pathways showing that the Nichoid causes alteration in genes strongly connected to mechanobiological functions. Moreover, we fully dissected this mechanism highlighting how the changes start at a membrane level, with subsequent alterations in the cytoskeleton, signaling pathways, and metabolism, all leading to a final alteration in gene expression. The results shown here demonstrate that the Nichoid influences the biological and genetic response of stem cells thorough specific alterations of cellular signaling. The characterization of these pathways elucidates the role of mechanical manipulation on stem cells, with possible implications in regenerative medicine applications.

## 1. Introduction

Nowadays, three-dimensional (3D) scaffolds are deeply investigated for their ability to guide cell fate [1,2]. In fact, biocompatible scaffolds have been developed to mimic the physiological microenvironment of specific cells and their growth inside these scaffolds typically allows a cellular response more representative of the in vivo behavior with respect to the 2D cell culture conditions [3,4,5,6]. For this reason, the use of 3D scaffolds is important in the field of regenerative medicine in order to improve stem cells therapeutic effect [7,8]. 

In particular, the “Nichoid,” a 3D scaffold inspired by the natural staminal niche, seems to have a great potential in the development of 3D cultures and could be of great relevance in the field of regenerative medicine [9,10,11] (Carelli et al. 2020, accepted in *Nanotheranostics*). This concept of a synthetic engineered niche is based on an elementary and precise geometry of the pores [10,11,12,13]. Thanks to its geometry and dimension, the Nichoid mimics the microarchitecture and the microenvironment of the native stem niche [14]. The cells grown inside the Nichoid are subjected to isotropic stimuli, which influence the spatial distribution of the cytoskeleton. It is these mechanical forces alone, without specific chemical supplementation to the medium, which strongly influence the cellular response [15]. Even so, the specific molecular components, which enact these responses, are yet to be characterized. In their specific physiological situation, cells are subjected to several external forces, such as fluid shear stress, osmotic forces, mechanical load, and stretch [16]. Moreover, cells have the ability to actively sense the external microenvironment that influences the morphology of membranes, the intracellular organization, their proliferation, and their migration [16,17]. Specifically, recent analysis on the neural stem cells niche have highlighted an increased stiffness in this part of the brain [18].

Mechanotransduction is the conversion of mechanical inputs into intracellular biochemical signals for the regulation of cellular physiology. It generally occurs at the cell–extracellular matrix (ECM) interface and after cell–cell contacts [16,19]. The cytoskeleton is a key player in this process as it is a protein-based network constituted principally by actin, microtubules, and intermediate filaments that extend throughout the cytoplasm [14,20]. The traction forces exerted by the connection between the cytoskeleton and the external microenvironment are transmitted to the nucleus through intracellular pathways. This results in a response in which forces trigger biochemical transduction that affects cell physiology and in particular, the synthesis of specific transcription factors in the nucleus [21]. Moreover, these mechanical stimuli can cause signaling cascades that also involve changes in gene expression [22,23] and the cellular metabolism [24].

Several studies have investigated the mechanical properties of different substrates and the consequent cellular responses, including proliferation, differentiation, and cell morphology [14,17,25]. For example, stiff substrates to promote osteogenesis [26] and soft biomaterials have been used to simulate the neural microenvironment [27]. In this context, Ortinau et al. seeded human neural progenitor cells in different concentrations of hydrogels-based matrix PuraMatrix to study the effects of the different concentrations on the assembly of the matrix and the subsequent influence on cells differentiation. In the same way, the external mechanical stimuli influence the growth and the biological response of stem cells [11,14]. The different analyses performed so far on cells grown inside the Nichoid were exclusively focused on cell morphology and imaging techniques [9,10,13,15], but an extensive analysis of the genetic perturbation happening in cells grown inside the Nichoid has not been yet carried out. The use of high-throughput technologies offers new and efficient strategies to perform a broad analysis of transcriptome to improve the knowledge of specific gene expression. 

Here, we investigated the Nichoid’s effect on neural precursors stem cells (NPCs), already investigated for their therapeutic efficacy in experimental animal models of neurodegenerative diseases such as Parkinson’s disease (PD) and in traumatic spinal cord injury (SCI) [28]. Indeed, transplantation of NPCs in preclinical models is feasible and safe [29,30,31,32,33,34,35]. NPCs, isolated from SVZ after the donor’s death, intrastriatally infused in a preclinical experimental model of PD promoted a rapid improvement of both animal motility performances and the expression of dopaminergic markers associated to a potent anti-inflammatory effect [29,30,32]. Moreover, the intravenous administration of NPCs in a preclinical animal model of traumatic SCI improved hind limb functional recovery, axons regenerations, and differentiation into cholinergic neuron cells [31,33,34,35]. The safety and efficacy of NPCs as cell therapy was investigated not only in preclinical animal models but also in human patients [36]. In this context, Madrazo et al. transplanted into the putamina of Parkinsonian patients human NPCs obtained from first trimester human fetal tissue. These cells expressed Oct-4 and Sox-2 and were tested preclinically for morphological and behavioral responses. NPCs suspensions were then injected into a group of eight patients. One year after cell grafting, all but one of the seven patients completing the study showed various degrees of motor improvement, and five of them showed better response to medications. Moreover, none of the patients showed unwanted motor disturbances, tumor formation, or any immune responses to the grafted cells, demonstrating the safety and efficacy of this transplantation [36]. 

In this work, we aim to dissect the effect of the 3D Nichoid scaffold on the transcriptome of NPCs, highlighting significant changes in the mechanotransduction process.

## 2. Results

### 2.1. Neural Precursor Cells (NPCs) Expanded inside the Nichoid Present with Different Cellular Organization and Growing Capabilities

**Figure 1:**
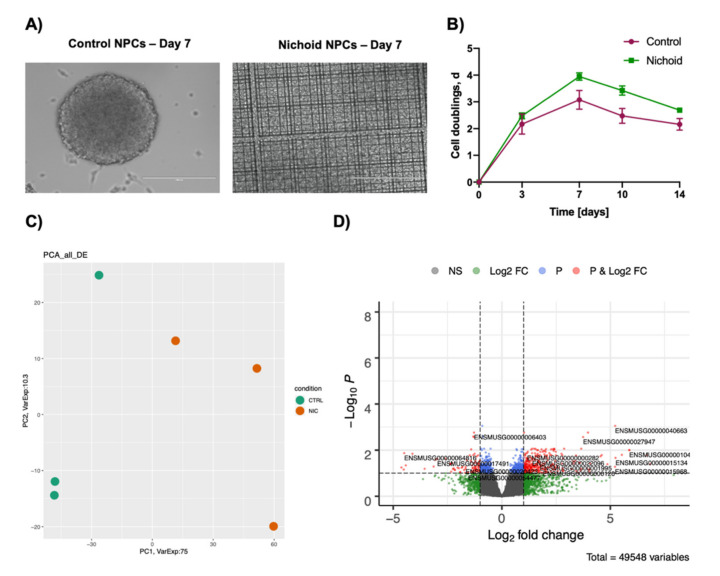
Neural precursors cells grown inside the Nichoid present with different morphology and transcription profiles. (**A**) In vivo direct light images (EVOS FL microscope, EuroClone) of NPCs neurospheres maintained in stem cells medium in standard floating conditions (control NPCs) or grown inside the Nichoid (Nichoid NPCs) at day 7. Scale bar: 200 μm. Images are representative of what was observed in more than 10 experiments. (**B**) Cell doublings were calculated after proliferation assay was performed by plating 10,000 NPCs either in standard floating condition or inside the Nichoid. Cells were counted and collected after 3, 7, 10, and 14 days in two experiments (*n* = 2). (**C**) For RNA-Seq, three samples for condition were analyzed (*n* = 3). Specifically, three experiments were performed each including one sample of NPCs grown in standard floating conditions for 7 days and one sample of NPCs grown inside the Nichoid for 7 days. The graph shows the principal component analysis (PCA) of differently expressed genes in NPCs grown on the Nichoid and in standard conditions. We considered as differentially expressed only genes showing |log_2_ (Nichoid samples/control samples) | ≥ 1 and a false discovery rate ≤ 0.1. (**D**) Volcano plot showing deregulated genes between NPCs grown on the Nichoid and in standard conditions.

NPCs were grown for 7 days both in standard floating conditions [28,31,33] and inside the Nichoid. At the end of this period, cells presented a different 3D organization and morphology (Figure 1A,B). Specifically, NPCs grown in standard floating conditions formed spheroids with a diameter dimension ranging from 100 to 400 μm (Figure 1A and Figure S1A), as previously reported [28]. On the contrary, NPCs expanded inside the Nichoid organized themselves in a carpet-like structure, expanding inside the niches (Figure 1A and Figure S1A). Moreover, cell grew exponentially in both the investigated conditions, Nichoid-grown NPCs, and control NPCS, reaching a peak at day 7, as shown by the doubling curve. Specifically, cells were detached from the scaffold and counted at day 3, 7, 10, and 14 and doubling times were calculated (see M&M section for further details) (Figure 1B). The cells present a reduction in proliferation after day 7 and for this reason, cells expanded for 7 days were chosen for subsequent experiments. It is worth noticing that Nichoid-grown NPCs present a higher number of cell doublings at all investigated time points. The cells also resulted compatible with the scaffolds, presenting with a 97.12% ± 0.02% viability in Nichoid-grown NPCs vs. 92.23% ± 1.10% in control-grown NPCs (Figure S1B).

Together, these observations highlighted strong morphology alterations, suggesting that the Nichoid scaffold leads to a different cellular organization compared to floating culture.

### 2.2. Deep Sequencing RNAs Expression Profiles in NPCs Grown inside the NICHOID vs. Control Conditions 

To investigate the pathways through which the Nichoid exerts its effects on NPCs, we performed a whole transcriptome analysis of NPCs grown in standard floating conditions or inside the Nichoid for 7 days. We detected a large number of differentially expressed coding and noncoding RNAs (DE RNAs) in NPCs grown inside the 3D scaffold with respect to standard conditions. PCA analysis of the DE RNAs in NPCs grown inside the Nichoid (Figure 1C) showed different expression profiles, suggesting that the Nichoid may have an important impact on many cellular features. A total of 1934 DE RNAs were identified, 81% (1577 out of 19,344) were coding genes (Figure 1D, Table 1, and Table S1). 

Among the top 10 deregulated coding genes we found RNAs involved in different pathways, e.g., cell adhesion and morphology, DNA binding and transcription, amino acid transport, enzymatic activity correlated with cellular metabolism, and also neuronal specification (Table 2). The significant number of deregulated genes and the implications that the most deregulated ones seem to have in key cellular processes could implicate a fundamental role for the Nichoid in the alteration of the cell’s biology.

### 2.3. Role of the Noncoding Transcriptome

Transcriptomic analysis revealed an alteration in noncoding RNAs expression. A total of 357 noncoding RNAs were found deregulated. A detailed classification of the functionally present ncRNAs is reported in Table 3; besides TEC class that are regions that require experimental validation for the presence of protein coding genes, the most represented functional class is that of lincRNAs with 60 deregulated genes (40 upregulated and 20 downregulated). LincRNAs are noncoding transcripts that localize in intergenic regions, and amongst the functional deregulated lincRNAs, there are genes correlated with pluripotency, cell survival, and gene expression. 

In particular, we found five downregulated lincRNAs (2900076A07Rik, Gm16892, Gm4262, Gm807, and C130071C03Rik) associated to pluripotency. Specifically, 2900076A07Rik, Gm16892, Gm4262, and Gm807 activation has been correlated to reprogramming of mouse fibroblasts [37], while C130071C03Rik facilitates neuronal differentiation sponging miR-101a-3p in mouse hippocampal tissue [38]. 

Gm26917 was upregulated in NPCs grown inside the Nichoid and it has been shown to promote proliferation and survival of muscle satellite cells acting as a competing endogenous RNA (ceRNA) for a proapoptotic miRNA, miR-29b [39].

Finally, we found two deregulated lincRNAs induced by p53, i.e., Lncpint and Linc-p21. Lncpint interacts with the polycomb repressive complex 2 [40] and Linc-p21 interacts with hnRNP-K [41]. Both these lncRNAs cause global gene repression in response to p53 control. Moreover, linc-p21 has been found to be deregulated in NPCs differentiation [42]. 

### 2.4. Pathway Analysis of Deregulated Transcripts

**Figure 2:**
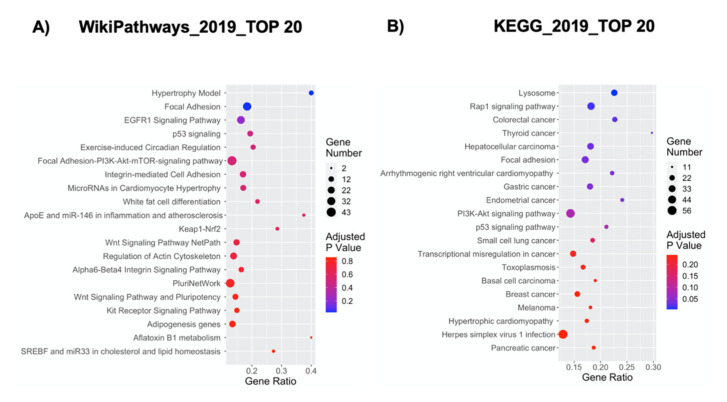
Pathway analysis for DE genes in NPCs expanded in the Nichoid compared to NPCs grown in standard conditions. The y-axis represents the name of the pathway, the x-axis represents the gene ratio, dot size represents the number of different genes, and the color indicates the adjusted *p*-value. (**A**) Dot plot of top 20 deregulated pathways from WikiPathways analysis. (**B**) Dot plot of top 20 deregulated pathways from KEGG analysis.

The deregulated transcripts with a deregulation ≥1 in terms of |Log_2_FC| were subjected to pathways analysis via the Enrichr web tool [43]. We identified the top 20 deregulated pathways with the WikiPathways 2019 and KEGG 2019 tools (Figure 2). Thanks to these analyses, we were able to highlight the pathways mainly altered when NPCs were grown inside the Nichoid. The 3D scaffold implicates changes in the cellular morphology and signaling, which could finally result in an overall deregulation of gene expression. The WikiPathways and KEGG analysis support this, as in the WikiPathways 2019, amongst the top 20 deregulated pathways (Figure 2A), we found five pathways implicated in alteration in the membrane and cytoskeleton conformation, supporting the morphological changes observed with in vivo microscopy, such as the close interactions with the scaffold. These pathways are: the focal adhesion pathway, the focal adhesion-PI3K-Akt-mTOR signaling pathway, the integrin-mediated cell adhesion pathway, the regulation of the actin cytoskeleton, and the alpha 6-beta 4 integrin signaling pathway. Other signaling pathways result strongly implicated, and these are the EGFR1 signaling pathway and the kit receptor signaling pathway. Changes in gene expression obtained simply after the cells are placed in the scaffold implicate pathways correlated with the cellular metabolism (e.g., white fat cell differentiation, keap1-nrf2, adipogenesis genes, aflatoxin b1 metabolism, SREBF and miR-33 in cholesterol, and lipid homeostasis) and proliferation (p53 signaling, hypertrophy model, miRNA in cardiomyocyte hypertrophy, Wnt signaling pathway netPath, pluriNetWork, Wnt signaling pathway, and pluripotency). A full list of the 149 deregulated WikiPathways 2019 is reported in Table S2. These findings are supported by KEGG 2019 analysis, which also presents pathways implicated in cellular morphology (e.g., focal adhesion), signaling pathways (e.g., Rap1 signaling pathway and PI3K-Akt signaling pathway) and proliferation (e.g., p53 pathway) (Figure 2B). The full list of the 277 significantly deregulated KEGG pathways is reported in Table S3. 

### 2.5. GO Terms Enrichment Show Significant Deregulation of Genes Implicated in Mechanotransduction via the Nichoid Scaffold

**Figure 3:**
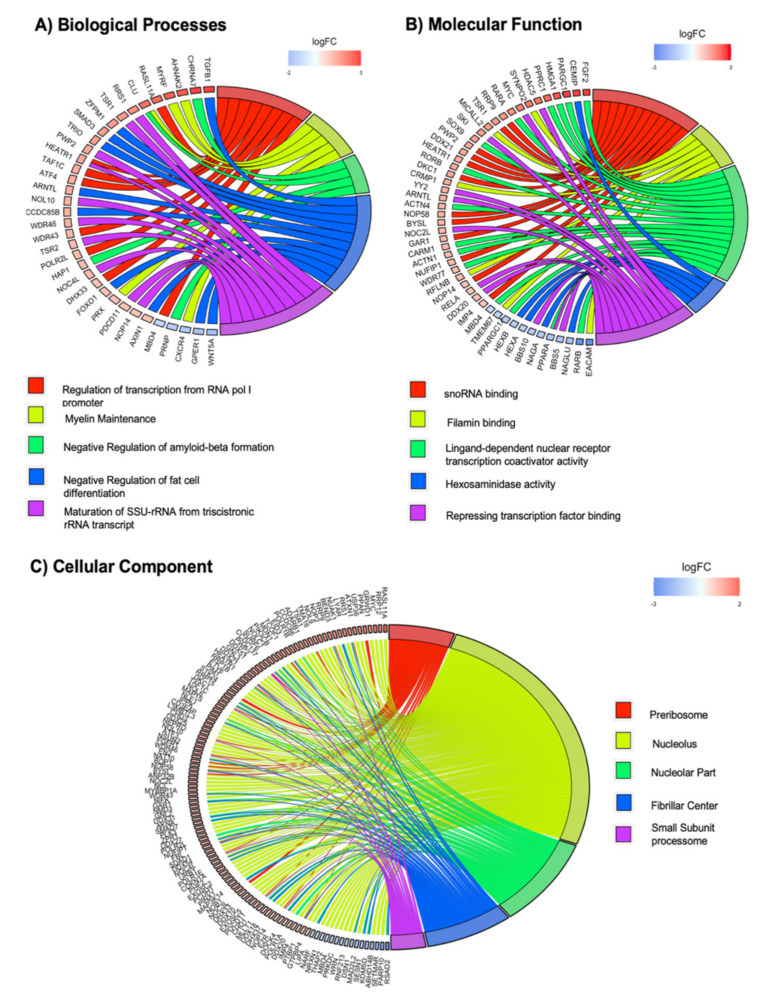
GO analysis for DE genes in NPCs expanded in the Nichoid compared to NPCs grown in standard conditions. (**A**) Top five enriched GO terms for Biological Process, (**B**) Molecular Function, and (**C**) Cellular Component are shown. The panels have been obtained considering all DE genes, and the color represents different pathways implicated to the deregulated genes.

Gene expression profiles of NPCs grown inside the Nichoid vs. control floating conditions were analyzed for GO terms enrichment in Biological Processes, Molecular Function, and Cellular Component (Figure 3). In particular, the deregulated transcripts with a deregulation ≥1 in terms of |Log2FC| were subjected to pathways analysis via the Enrichr web tool [43]. GO categories for each function were sorted by increasing *p*-value. When considering the top 5 GO terms, we identified 203 deregulated genes (35 for Biological Process, 48 for Molecular Function, and 120 for Cellular Component). With respect to the Biological Processes, we identified a total of more than 3800 pathways (Table S4). Among the top 5 GO terms, we found pathways involved in transcription regulation, myelin maintenance, regulation of cell differentiation, and necrosis (Figure 3A). Tgfb1 and Wnt5a are, respectively, the most up- and downregulated genes with a role in Biological Process. Tgfb1 is a coding gene for a secreted ligand of the TGF-β superfamily of proteins. It regulates the growth and differentiation of various cell types and is involved in various processes, such as normal development, immune function, microglia function, and responses to neurodegeneration [44,45]. Interestingly, cytoskeletal tension has been demonstrated to regulate TGF-β’s signaling [46,47]. Vil1 encodes a member of a family of calcium-regulated actin-binding proteins. It involved in epidermal growth factor receptor signaling pathway, which plays an important role in cell proliferation, differentiation, and migration [48].

With the GO Molecular Function, we identified 800 significantly deregulated pathways (Figure 3B and Table S5). Among them, the most enriched terms we found were “snoRNA binding,” “filamin binding,” and “ligand-dependent nuclear receptor transcription coactivator activity.” The deregulation in transcriptional activity and response suggests that the Nichoid exerts its effects through a selective transcriptional activation. Actin binding is also present, supporting the implication of cytoskeleton remodeling. Fgf2 and Ceacam1 are, respectively, the most up- and downregulated genes in Molecular Function. Fgf2 is a member of the fibroblast growth factor family, which encodes proteins involved in limb and nervous system development. Ceacam1 encodes for a cell adhesion molecule and is involved in filamin binding, where filamins are important actin-binding proteins that regulate cytoskeleton remodeling [49]. 

With respect to Cellular Component, we found 314 pathways (Figure 3C and Table S6). The top three GO Cellular Component terms were “preribosome,” “nucleolus,” and “nucleolar part.” Moreover, the analysis revealed a strong implication for the contractile Cellular Component, highlighting actomyosin, cytoskeleton, stress fibers, and contractile actin filament bundle. This demonstrates, again, that the maintenance inside the Nichoid strongly alters the cytoskeleton morphology, thus leading to an altered transcriptional cascade (Figure 3C). Rasl11a and Rsad2 are, respectively, the most up- and downregulated genes in Cellular Component. Rasl11a is a member of GTPase protein family and modulates a variety of cellular effects such as growth control, cytoskeletal rearrangements, cell survival, and apoptosis [50]. Rsad2 is a multifunctional protein in viral processes that can inhibit many DNA and RNA viruses, and it is involved in more than one process, such as nucleolar part and nucleolus.

These significantly enriched terms provide a lot information to further understand the role that deregulated genes play in Nichoid structure.

### 2.6. NPCs Grown inside Nichoid Differently Express Cytoskeleton and Membrane Organization-Related Genes

In specific biological contexts, cells are continuously subjected to a large variety of mechanical forces, which play a critical role in regulating their functions by either activating or tuning signal transduction pathways [51,52]. These forces can be sensed by cell adhesion surface receptors, such as integrins and cadherins, that are strongly connected to the cytoskeleton, resulting in alteration of cell membrane [24,51,52]. An example of this mechanism is exerted by the RhoA signaling pathway, a small GTPase protein, that plays an important role in regulating actin cytoskeleton and its response to mechanical stimuli. When active, RhoA stimulates the Rho kinase, which promotes contractility and bundling of actin filaments [51]. Indeed, our gene expression analysis reveals the presence of deregulated Rho genes (specifically, RhoU, and RhoJ were found upregulated) (Table S1). RhoU is a GTPase encoded by Rhou gene that mediates cell morphology, cytoskeletal organization, and proliferation [53]. RhoJ is an endothelially expressed member of the Cdc42 (cell division cycle 42) subfamily that has been shown to be a focal-adhesion-localized Rho GTPase and that can modulate focal adhesion number and actomyosin contractility [54].

**Figure 4:**
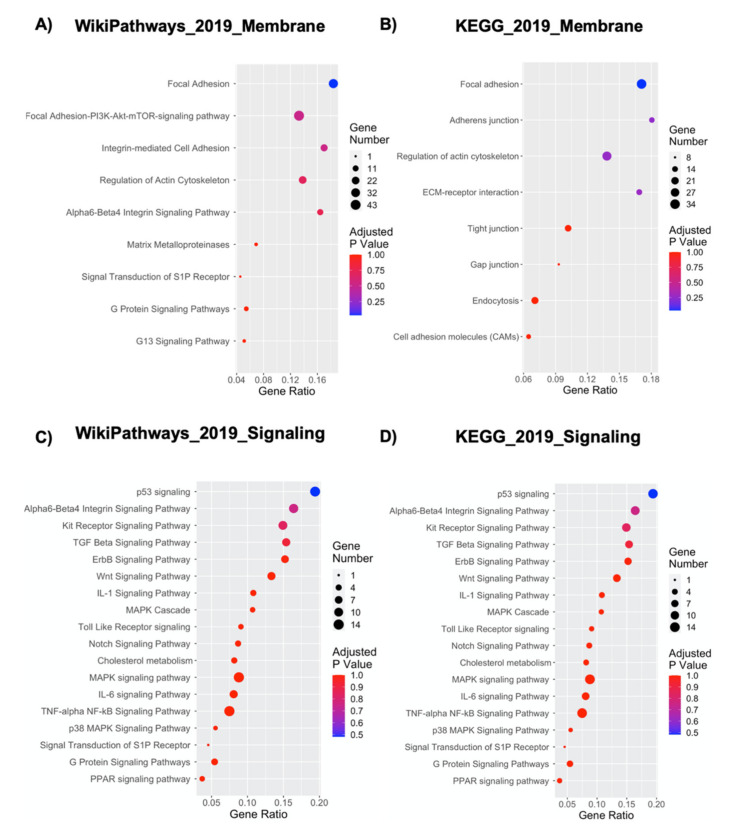
Deregulated pathways and genes correlated with membrane and signal transduction processes. WikiPathways analysis (**A**) and KEGG analysis (**B**) highlighted nine and eight deregulated pathways, respectively, for membrane processes. WikiPathways analysis (**C**) and KEGG analysis (**D**) highlighted 18 and 29 deregulated pathways, respectively, for signal transduction. The y-axis represents the name of the pathway, the x-axis represents the Rich factor, dot size represents the number of different genes, and the color indicates the adjusted *p*-value.

The interaction between NPCs and the Nichoid scaffold results in alteration of cell organization, suggesting a possible modification of cellular membrane and, as a consequence, also a different cytoskeletal organization. Figure 4 shows a link between NPCs growth inside Nichoid and a strong alteration in membrane-associated pathways. Indeed, 9 out of 149 WikiPathways 2019 and 8 out of 277 KEGG 2019 pathways were correlated with membrane and cytoskeleton alteration (Figure 4A,B). focal adhesions, adherens junctions, regulation of actin cytoskeleton, ECM-receptor interaction (KEGG pathways), focal adhesion-PI3K-Akt-mTOR-signaling pathway, integrin-mediated cell adhesion, regulation of actin cytoskeleton, and alpha 6-beta 4 integrin signaling pathway have emerged as most deregulated pathways. Focal adhesions are large macromolecules complexes, which connect cell cytoskeleton to ECM, and can be influenced by mechanotransduction through protein domains that undergo conformation changes, resulting in regulation of cell spreading, shape, and migration [55]. 

Another important class of cell adhesion’s proteins whose genes are affected by the Nichoid structure are integrins and cadherins. These proteins are responsible for adhesion junctions: integrins mediate adhesion between the cells and ECM, and cadherins mediate adhesion between cells. The coordinated interaction between integrins and cadherins mechanically connects the actin cytoskeleton to neighboring cells and the matrix in order to regulate multicellular processes [56]. KEGG analysis reveals the presence of pathways related to cell–cell interaction, such as tight junction, Gap junction, and ECM-receptor interaction, which have emerged as deregulated pathways and indicate an altered intracellular interaction in Nichoid-grown NPCs. Gene expression analysis also shows that specific genes encoding for integrins have been found deregulated. Itga6, Itga2b, Itga9, and Itga5 emerged as upregulated, whereas Itga4 and Itgb8 emerged as downregulated (Table S1). Moreover, many of these genes have been found deregulated in more than one pathway both in KEGG and in WikiPathways, highlighting Nichoid’s influence on cellular organization.

Lastly, signaling pathways linked to membrane alteration have emerged as deregulated pathways in WikiPathways analysis (G protein signaling pathways, G13 signaling pathways, signal transduction of S1P receptor). These pathways are related to the action of G-proteins and G-protein coupled receptors (GPCRs), involved in transmission of signals from outside to inside cells and in cellular signal transduction [57]. The bioactive lipid sphingosine-1-phosphate (S1P) binds G-proteins regulating different biological processes: proliferation, apoptosis, cellular differentiation, development, and angiogenesis [57,58]. These results demonstrate that the Nichoid structure strongly impacts cytoskeleton organization and cell–cell interactions, also regulating downstream signaling pathways.

### 2.7. Cellular Growth inside the Nichoid Influences Genes Implicated in Signaling Transduction Pathways of Mechanotransduction

Several studies concerning mechanotransduction have shown that conformational alterations in cell behavior are not only triggered by the link between membrane-bound integrins and adhesive ligands but also are related to subsequent biochemical signal transduction, which can also alter specific nuclear processes [19]. Figure 4C,D shows the deregulated pathways related to cell signaling in the mechanotransduction process of NPCs grown inside the Nichoid. In brief, 18 out of 149 WikiPathways 2019 and 29 out of 277 KEGG 2019 pathways were correlated with this cell signaling process. 

Different kinase pathways, reported to be deregulated in Nichoid-grown NPCs (Figure 4C,D), such as MAPK cascade, MAPK signaling pathway, PI3K-Akt signaling pathway, and AMPK signaling pathway, are the most well-known pathways related to mechanotransduction. They are involved in several responses, in particular, they connect the plasma membrane with cytoplasmic and nuclear events [59], controlling a large number of fundamental cellular processes including growth, proliferation, differentiation, motility, stress response, survival, and apoptosis [60]. Several genes involved in these pathways are upregulated in cells grown inside the Nichoid with respect to the control condition; in the MAPK signaling pathway, we report 12 genes upregulated (dusp4, tgfb1, mras, il1r1, myc, mink1, arrb1, kras, map3k14, gck, atf4, and hspa1a; Table S1) from WikiPathways analysis and 41 upregulated genes from KEGG, suggesting an increase in this pathway’s activity in Nichoid-grown NPCs (Figure 4C,D). Another important deregulated pathway involved in cell signal transduction is the Wnt signaling pathway, which regulates crucial aspects of cell fate determination, cell migration, cell polarity, neural patterning, and organogenesis during embryonic development [61]. 

In KEGG analysis, the most significantly deregulated pathway is the Rap1 signaling pathway, a key modulator of integrin- and cadherin-regulated processes involved in mechanotransduction [62]. In this specific pathway, 38 genes result deregulated, suggesting an important difference between the Nichoid growth and the standard condition (Figure 4C,D). Indeed, the integrin signaling pathway (alpha 6-beta 4 integrin signaling pathway, Figure 4C,D) also has a significant impact on signaling molecules that stimulate migration [63]. 

Moreover, more specific deregulated pathways (p53, p38, and TGF-β; Figure 4C,D) are also involved in the process of mechanotransduction but are not as fully characterized in this context. We found that the most deregulated pathway in WikiPathways 2019 was the p53 signaling that regulates cell cycle, differentiation, metabolism, and transcription process [64]. Regarding the TGF-β signaling pathway, recent advances in cellular mechanobiology highlight the role of transforming growth factor-beta (TGF-β) in mediating cellular responses to physiological cues [46,47]. 

### 2.8. NPCs’ Growth inside the Nichoid Significantly Impacts Cellular Metabolism-Related Genes

**Figure 5:**
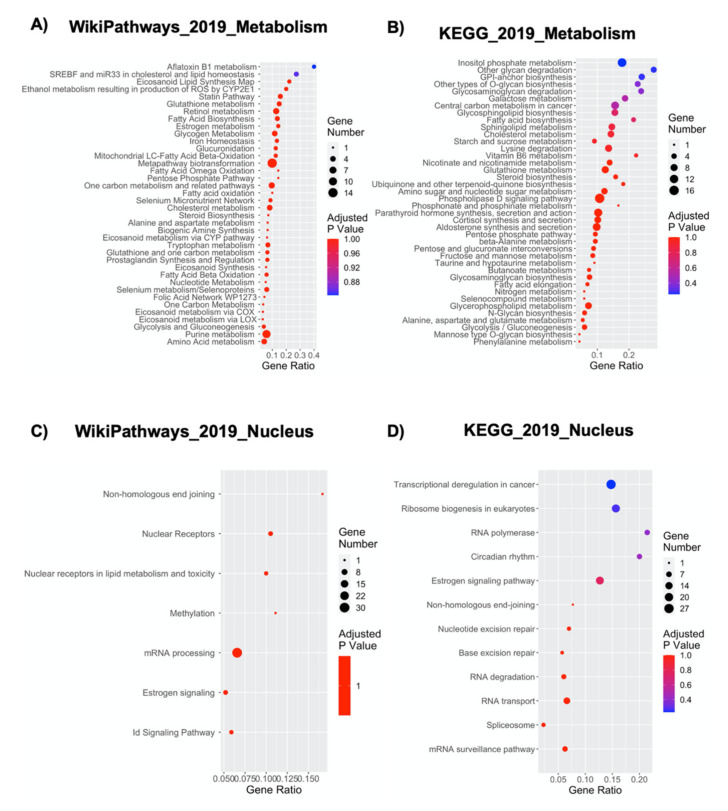
Deregulated pathways and genes correlated with metabolic alterations and nuclear response. WikiPathways analysis (**A**) and KEGG analysis (**B**) highlighted 38 and 68 deregulated pathways, respectively, for metabolic alterations. In panel (**B**) are shown the top 40 deregulated pathways. WikiPathways analysis (**C**) and KEGG analysis (**D**) highlighted 6 and 10 deregulated pathways, respectively, for nuclear response. The y-axis represents the name of the pathway, the x-axis represents the Rich factor, dot size represents the number of different genes, and the color indicates the adjusted *p*-value.

Cellular metabolism is typically defined as the sum of biochemical processes that either produce or consume energy [65]. These metabolic processes can be simplified in pathways involving three main classes of nutrients: carbohydrates, fatty acids, and amino acids, necessary for maintaining energy homeostasis [65]. A tight metabolic regulation is fundamental for the control of all cellular processes, but less is known about its specific link with mechanotransduction [24,66]. Figure 5A,B shows the effect of NPCs expansion inside the Nichoid on numerous metabolism-associated pathways. Indeed, 38 out of 149 WikiPathways 2019 and 68 out of 277 KEGG 2019 pathways were correlated with metabolic processes (Figure 5A,B and Table S7).

As the metabolic deregulation obtained in Nichoid-grown NPCs is very significant, it is possible to note an involvement in most classes of cellular metabolites, e.g., lipids, carbohydrates, proteins, vitamins, nucleotide metabolism, and even the Metapathway biotransformation (WikiPathways), which indicates a global metabolic deregulation (Figure 5A,B). The most significant alterations seem to be present in pathways controlling lipid homeostasis (16 WikiPathways and 17 KEGG pathways; Figure 5A,B and Table S7). This could have two possible implications: on the one hand, fatty acid biosynthesis, beta-oxidation, biosynthesis of unsaturated fatty, acids etc. are evidences of an alteration in the energetic demand and subsequent production, and on the other hand, pathways involving membrane lipidomics (e.g., sphingolipid metabolism) could result in an altered cellular signaling-transduction (Figure 5A,B). It is interesting to note that Lpin1 (which codifies for Lipin-1, Table S1) was found downregulated in the Nichoid-grown NPCs, as this gene, crucial for lipid synthesis and accumulation, has been demonstrated to be inhibited by reduced actomyosin contractility [67]. The second lipid-related aspect involves specific plasma membrane microdomains, enriched in glycosphingolipids, gangliosides, and sterols (such as cholesterol) to form membrane/lipid rafts (Figure 5A,B). The clustering of these rafts into more active signaling platforms can depend upon interactions with and dynamic rearrangement of the cytoskeleton. Subsequently, these rafts regulate cellular polarity, adherence to the extracellular matrix, signaling events, cell migration, mechanotransduction, and even neuronal growth and signaling [68]. Finally, there could also be a bidirectional regulation of mechanotransduction, induced by metabolic pathways such as sphingolipid biosynthesis (Figure 5A,B), through activation of the mechanical signaling downstream of integrins, including RhoA [69]. 

Another class of molecules that appear to be significantly influenced by the Nichoid are carbohydrates. The WikiPathways analysis shows a deregulation in 4 pathways related to carbon metabolism (glycogen metabolism, pentose phosphate pathway, one carbon metabolism and related pathways, and glycolysis and gluconeogenesis), whereas KEGG shows up to 17 carbon-related altered metabolic pathways (amongst these, the four reported in WikiPathways are glycosaminoglycan degradation, galactose metabolism, starch and sucrose metabolism, and several others) (Figure 5A,B). A possible explanation for this deregulation could be found in the fact that activation of E-cadherin simply through mechanical forces stimulates the AMP-activated protein kinase (AMPK) activation, a master regulator of mammalian metabolism [70]. Active AMPK stimulates energy generating processes (such as glucose uptake) and decreases those processes that are energy consuming (glycogen synthesis) [71]. It is very interesting to observe that amongst the genes upregulated in Nichoid-grown NPCs, we found Prkab2, which codes for a regulatory subunit of AMPK (Table S1).

Lastly, amino acids-related metabolic pathways are also influenced by the Nichoid (6 in WikiPathways and 13 in KEGG), but no specific works have so far specifically implicated mechanotransduction and their specific metabolism. Lastly, a deregulation is found also in metabolic pathways involving vitamin (retinol metabolism, folic acid network, and vitamin B6 metabolism) and nucleotides biosynthesis (nucleotide metabolism, one carbon metabolism, pyrimidine metabolism, and purine metabolism), suggesting that the Nichoid impacts broadly the metabolism, regulating also metabolic pathways, which have not yet been correlated strictly to mechanotransduction (Figure 5A,B).

### 2.9. The Nichoid Scaffold Influences Nuclear Compartment Processes through Alteration of Related Gene Expression

The idea that mechanical signals influence the expression of specific mechanosensitive genes has been proposed some years ago [72], but only recently, a number of reports showed that the nucleus has its own mechanosensitive apparatus [73]. Indeed, it has already been demonstrated that mechanotransduction can modulate the shape and the structure of the nucleus in response to changes in the interaction between cytoskeleton and nucleoskeleton [74]. Moreover, mechanical stimuli can affect chromatin reorganization [75], through the expression of specific genes involved in the maintenance of the nuclear structure and chromatin remodeling. An example of this mechanism is exerted by lamin A, which can modulate chromatin structure interacting directly with the DNA [76] or indirectly through other transcription factor such as Fos family members [77]. Interestingly, lamin A and 2 Fos family members (Fosl2 and Fosl) are deregulated in Nichoid-grown NPCs (Table S1). 

Indeed, among the enriched deregulated pathways, we found 6 and 10 nuclear related pathways from WikiPathways and KEGG 2019 analysis, respectively (Figure 5C,D). The emerged pathways concern different type of nuclear processes: DNA repair mechanisms, modulation of transcription, regulation of molecule transport through nuclear membrane, RNA metabolism, and finally, activation of specific mechanosensitive genes. 

Nonhomologous end-joining, nucleotide excision repair, and base excision repair (KEGG pathway) and Nonhomologous end joining (WikiPathways) have emerged as deregulated pathways (Figure 5C,D). Together, these pathways point out alterations in DNA repair mechanisms, both involving double- or single-strand break, in response to the Nichoid-growth. Components of the nucleoskeleton, which is prone to mechanical stimuli, have been related to homologous recombination and DNA base excision repair [78,79]. 

Modulation of transcription is also affected by the Nichoid scaffold. Transcriptional misregulation in cancer, RNA polymerase (KEGG pathway), methylation, nuclear receptors, nuclear receptors in lipid metabolism, and toxicity and ID signaling pathway (WikiPathways) highlighted changes in modulation of transcription at different levels (Figure 5C,D). We found alteration in six RNA polymerase subunits (Polr2l, Polr1a, Polr3d, Polr1b, Polr1e, and Polr3e, Table S1) suggesting a change in RNA polymerase activity, which has already been observed in response to mechanotransduction [80]. Epigenetics mechanisms control and modify gene expression and can be activated by numerous factors including mechanical stimuli [81]. Interestingly, HADC5 and HDAC11, two members of the HDAC family, one of the most involved enzymes in epigenetics regulation, were deregulated in response to the Nichoid (Table S1). In addition, transcription factors respond to mechanotransduction [82], for instance, we found deregulation in nuclear receptors, nuclear receptors in lipid metabolism, and in ID signaling pathway which can influence the activity of specific transcription factors leading to global changes in RNA transcription (Figure 5C,D).

We also found RNA transport to be one of the deregulated KEGG pathways (Figure 5C,D). The transport of RNA from the nucleus to the cytoplasm is essential for gene expression, and it is mediated by the nuclear pore complex composed of nucleoporins [83], some of which were found deregulated (Ipo4, Nupr1, and Nup62, Table S1). RNA transport and its localization have already been associated to mechanotransduction; depending on the stiffness of extracellular matrix, RNA is relocated and mediates different processes [84]. 

Finally, we observed alterations in RNA metabolism: Ribosome biogenesis in eukaryotes, spliceosome, mRNA surveillance pathway, RNA degradation (KEGG pathway), and mRNA processing (WikiPathways) (Figure 5C,D). Ribosomal biogenesis, i.e., ribosomal RNA and ribosome-associated proteins, occurs both in the cytoplasm and in the nucleus, and it also involves nuclear transport mechanisms. Interestingly, we found upregulated an importin (Ipo4) that specifically mediates the import of ribosomal protein (Table S1). In skeletal muscles, the upregulation of factors required for ribosomal biogenesis has been related to increased mechanical activity [85]. Spliceosome, mRNA processing, mRNA surveillance pathway, and RNA degradation are processes that control every step of an RNA molecule life deeply affecting the cell fate (Figure 5C,D). Indeed, splicing and quality control mechanism can also be controlled by mechanotransduction [86,87].

### 2.10. Influence of the Nichoid on Cytoskeletal Organization and Adhesion Complexes Formation

**Figure 6:**
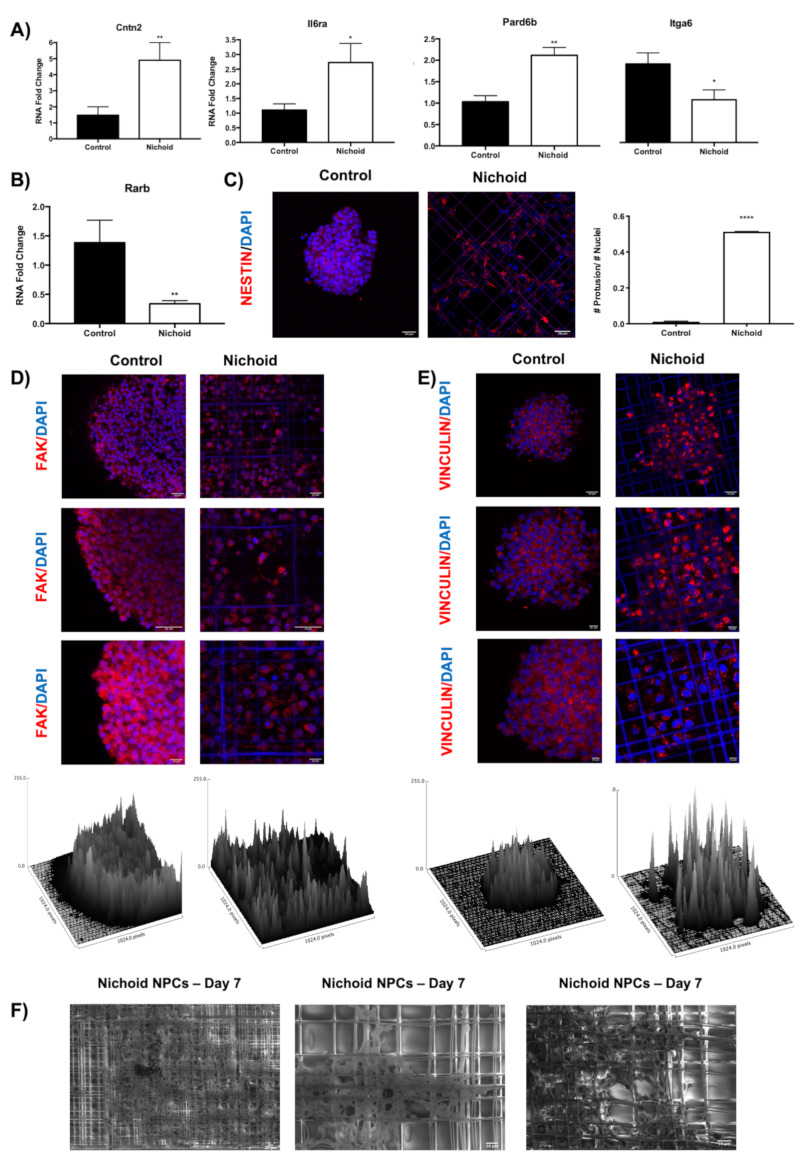
Evidences of adhesion complexes and cytoskeletal deregulation. (**A**) Differential expression of genes involved in adhesion complexes was verified by real-time PCR in a larger cohort (four experiments each performed in duplicates, N = 8) of NPCs grown in standard floating conditions and NPCs grown inside the Nichoid for Cntn2, Il6ra, Pard6b, and Itga6. GAPDH was used as housekeeping gene. Data are expressed as mean of four independent experiments, each performed in duplicate ± SEM (*n* = 8), * *p* < 0.05, ** *p* < 0.01 vs. control. (**B**) Differential expression of genes involved in cytoskeletal remodeling was verified by real-time PCR in a larger cohort (four experiments each performed in duplicates, N = 8) of NPCs grown in standard floating conditions and NPCs grown inside the Nichoid for Rarb. GAPDH was used as housekeeping gene. Data are expressed as mean of four independent experiments, each performed in duplicate ± SEM (n = 8), ** *p* < 0.01, vs. control. (**C**) Immunofluorescence images of Nestin, in red, and nuclei, in blue (DAPI), of standard floating NPCs or inside the Nichoid. Scale bar: 20 μm. Images are representative of two fields acquired per Nichoid (N = 2). The histogram refers to the number of prolongments counted in the image over the total nuclei number (N = 2, **** *p* < 0.0001 vs. control). (**D**) Immunofluorescence images of focal adhesion kinase (FAK), in red, and nuclei, in blue (DAPI), of standard floating NPCs or inside the Nichoid. Scale bar: 20 μm for the two top images and 10 μm for the bottom images. Images are representative of three fields acquired per Nichoid (N = 3). The surface plot was obtained using the Image J software, and it represents the distribution of the marker on the analyzed surface. (**E**) Immunofluorescence images of Vinculin, in red, and nuclei, in blue (DAPI), of standard floating NPCs or inside the Nichoid. Scale bar: 20 μm for the two top images and 10 μm for the bottom images. Images are representative of three fields acquired per Nichoid (N = 3). The surface plot was obtained using the Image J software, and it represents the distribution of the marker on the analyzed surface. (**F**) Images of NPCs grown for 7 days inside the Nichoid and then fixed and analyzed by environmental scanning electron microscope (ESEM) at 2000× magnification. Images are representative of six fields acquired per Nichoid, in 2 different Nichoids (*n* = 12). Scale bar: 10 µm for the left image and 10 μm for the right images.

The results obtained via RNA-Seq highlighted a strong perturbation in gene expression of adhesion complexes molecules, along with components of the cytoskeleton. Via real-time PCR, we analyzed in a larger cohort of samples (eight NPCs grown in standard conditions and eight NPCs grown inside the Nichoid), the expression of genes involved in adhesion complexes (Figure 6A) and cytoskeleton organization (Figure 6B). Specifically, Cntn2 was found upregulated in NPCs grown inside the Nichoid both in RNA-Seq (log_2_FC = 4.91) and via real-time PCR (Figure 6A). This gene encodes for the contactin 2 protein, part of the immunoglobulin superfamily of cell adhesion molecules [88]. The protein is a glycosylphosphatidylinositol (GPI)-anchored neuronal membrane protein, which plays a role in the proliferation, migration, and axon guidance [89,90]. Moreover, Il6ra, a protein coding gene associated with the PI3K-Akt signaling pathway and the focal adhesion-PI3K-Akt-mTOR-signaling pathway (WP2841), was found upregulated in NPCs grown inside the Nichoid (log_2_FC = 4.92) [91,92] (Figure 6A). Pard6b (Par-6 Family Cell Polarity Regulator Beta), a molecule implicated in cell polarity and tight junction formation [93,94], was significantly upregulated in Nichoid-grown NPCs by log_2_FC = 4.88. Interestingly, it was demonstrated that in Xenopus epidermis, Par6b autonomously regulates the dynamics of apicobasal polarity, as it is required to maintain the “basolateral” state in both epidermal layers. This supports the idea that the Nichoid recapitulates the characteristics of a Niche-like structure [95] (Figure 6A). Itga6 (integrin alpha 6) was found downregulated by log_2_FC= −1.56 in NPCs grown inside the Nichoid (Figure 6A). This gene encodes for a member of the integrin superfamily, transmembrane receptors involved cell adhesion, and signaling. This protein has been shown to heterodimerize with beta 4 to bind laminin and to form the main component of hemidesmosomes, which mediate attachment of epithelia to basement membranes [25,96]. Moreover, pertaining to cytoskeletal organization, Rarb (retinoic acid receptor beta) was found downregulated inside the Nichoid by log_2_FC = −4.6 (Figure 6B). Indeed, retinoic acid receptors (RAR), regulate mechanotransduction-related gene expression, upregulating the expression of genes such as the intermediate filament proteins lamin A and C and related to the increase of matrix elasticity [97]. To further support these observations, NPCs were labeled for Nestin, a type VI intermediate filament protein, expressed in the early stages of development in the central nervous system [98,99] (Figure 6C). It is possible to appreciate how Nestin’s distribution changes in Nichoid-expanded NPCs as opposed to those cultured in standard floating conditions. Indeed, in neurospheres, the intermediate filament protein, and subsequent cytoskeletal organization, surround the cells’ nuclei without protruding from the cell core. On the contrary, in Nichoid grown NPCs, 50% of the cells present protrusions, as reported in the histogram showed in Figure 6C. The formation of focal adhesion complexes was investigated through the labeling of focal adhesion kinase, a kinase found concentrated in focal adhesions present amongst cells and their surroundings, specifically between cellular and matrix components, and involved in mechanotransduction processes [100] (Figure 6D). The immunofluorescence analysis shows a different topological localization for the FAK in Nichoid-grown NPCs than in standard floating NPCs. Indeed, the surface plot reported in Figure 6D shows that in spheroids, the FAK is mainly expressed on the edges of the sphere, with a decreased intensity in the spheroid core. Interestingly, the distribution is different in NPCs grown inside the Nichoid, where the intensity of the signal is homogenous in scaffold-grown NPCs (Figure 6D). Moreover, NPCs were labeled for Vinculin, a membrane-cytoskeletal protein associated with both cell–cell and cell–matrix adhesions, and also strongly implicated in mechanotransduction processes [70] (Figure 6E). Vinculin is expressed in a similar manned in the whole spheroid, but its expression intensity is relevantly higher in Nichoid-grown NPCs, indicating again an increase in focal adhesion complexes formation (Figure 6E). To analyze the ultrastructure of scaffold-grown NPCs, cells grown inside the Nichoid were also analyzed by environmental scanning electron microscopy (ESEM) allowing for a high-resolution study (Figure 6F). With this approach, it was possible to appreciate that the NPCs cytoskeleton made protrusions strongly interacting with the engineered scaffold (Figure 6F).

### 2.11. Deregulation of Genes Involved in Regeneration and Neural-Related Processes Highlights Potential Translational Applications

**Figure 7:**
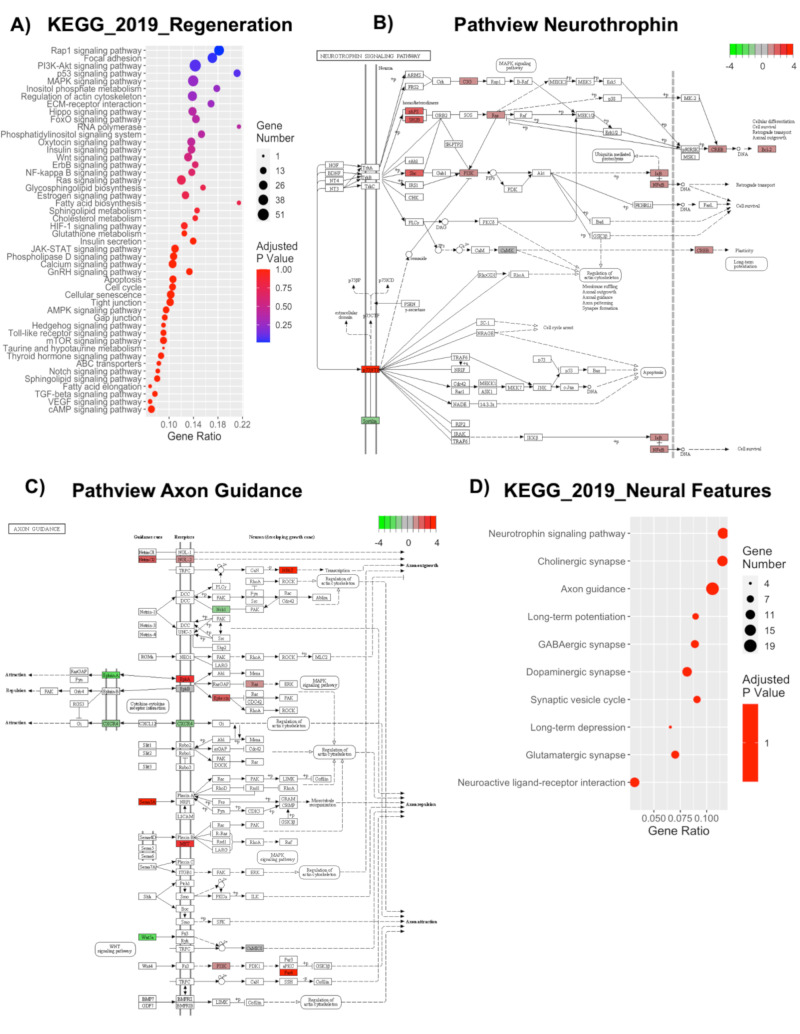
Deregulated pathways and genes correlated with regeneration and neural features. (**A**) KEGG analysis highlighted 48 deregulated pathways for regenerative processes. (**B**,**C**) Pathview of neurotrophins signaling pathway and axon guidance, respectively. In red, upregulated genes are shown, whereas in green, downregulated ones are shown. (**D**) KEGG analysis of neural features highlighted 10 deregulated pathways. The y-axis represents the name of the pathway, the x-axis represents the Rich factor, dot size represents the number of different genes, and the color indicates the adjusted *p*-value.

The results hereby reported highlight a significant transcriptional deregulation in mechanotransduction-reported processes. We, thus, wished to investigate if these effects could have potential translational applications and could be of possible relevance in the field of regenerative medicine. To do so, we decided to highlight the processes, which could indicate a regenerative potential and could be of relevance in neurological applications. Indeed, 48 out of 277 KEGG 2019 pathways were correlated with regenerative processes (Figure 7A). The PI3K pathway, which includes the highest number of deregulated genes [54], is known to play important roles in cell survival and has been implicated in brain repair after ischemic damage [101]. Other significant pathways, including a high number of genes and implicated in regeneration, are RAP1 signaling, MAPK signaling, Hippo signaling, FoxO signaling pathways, and even pathways implicated in cytoskeletal organization (e.g., focal adhesions, regulation of actin cytoskeleton, ECM receptor interaction, etc.). Moreover, we found several processes that suggest an enhancement in neural features (Figure 7B–D). One of the most relevant is the neurotrophins signaling pathway, which concerns a family of trophic factors involved in the differentiation and survival of neural cells [102]. Interestingly, 14 genes deregulated in Nichoid-grown NPCs were implicated in this process, and 13 of them were upregulated, including the nerve growth factor receptor gene, crucial in eliciting a neurotrophic response (Figure 7B). Another relevant pathway is that which concerns axon guidance, a key stage in the formation of neuronal networks [103]. In this case, 19 implicated genes were deregulated in Nichoid-grown NPCs, with the upregulated molecules leading to axon outgrowth and enhancement and the downregulated ones coding for genes implicated in axon repulsion (Figure 7C). This computational analysis could suggest an increased expression of genes facilitating axon guidance. Overall, 10 out of 277 KEGG 2019 pathways were correlated with neural enhancing features, including synapses formation, synaptic cycling, and synaptic plasticity (Figure 7D).

### 2.12. Nichoid Grown NPCs Are Able to Differentiate to a Neuronal Phenotype 

**Figure 8:**
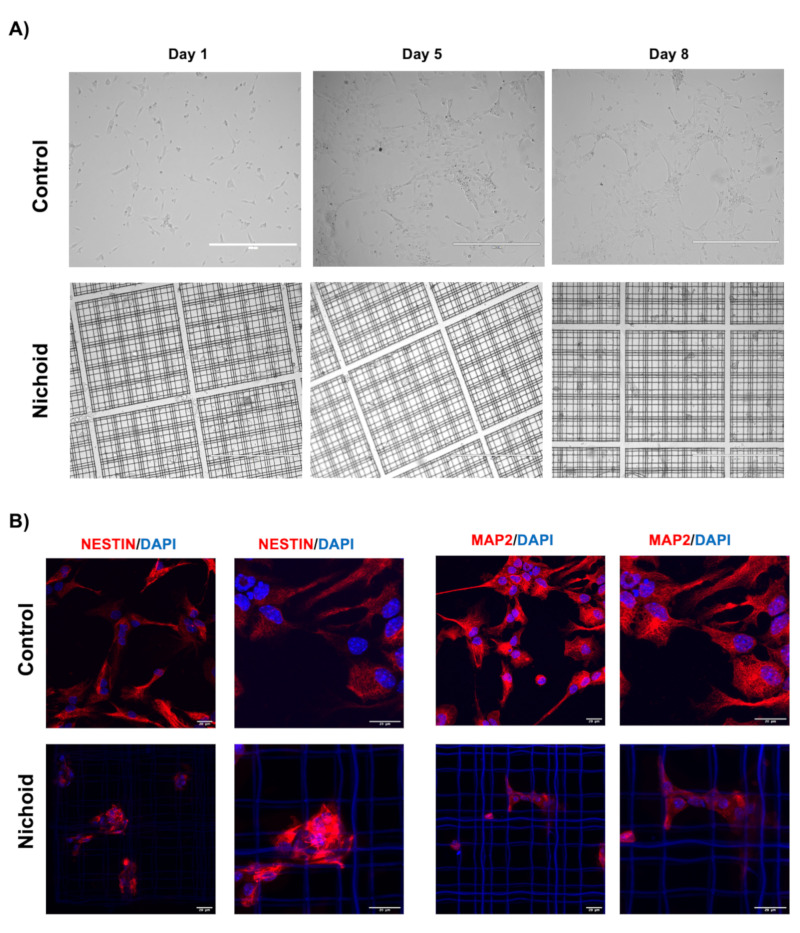
Differentiated neural precursors cells grown inside the Nichoid. (**A**) In vivo direct light images (EVOS FL microscope, EuroClone) of control NPCs (control) or differentiated inside the Nichoid (Nichoid) stimulated with differentiation medium at day 1, 5, and 8. Scale bar: 400 μm. Images are representative of what was observed in two independent experiments (**B**) Immunofluorescence images of NESTIN and MAP2, in red, and nuclei, in blue (DAPI), of differentiated NPCs in control flat conditions or inside the Nichoid. Scale bar: 20 μm. Images are representative of three fields acquired per Nichoid (*n* = 3).

To investigate the differentiation potential of NPCs grown inside the Nichoid, we applied an in vitro differentiation chemical protocol, which requires a specific differentiation medium (please see M&M section for further details) [28,99]. An interesting aspect is that NPCs grown in control conditions always require the Matrigel substrate in order to adhere, however, Nichoid-grown NPCs are able to differentiate without the organic substrate (Figure 8A). We performed an immunofluorescence analysis for MAP2 and Nestin to investigate the expression of differentiation markers, and we observed that Nichoid expanded NPCs are capable to differentiate to a neuronal phenotype (Figure 8B).

### 2.13. Deep Sequencing RNAs Expression Profiles and Pathways Analysis in NPCs Replated after Nichoid-Expansion vs. Control Conditions

**Figure 9:**
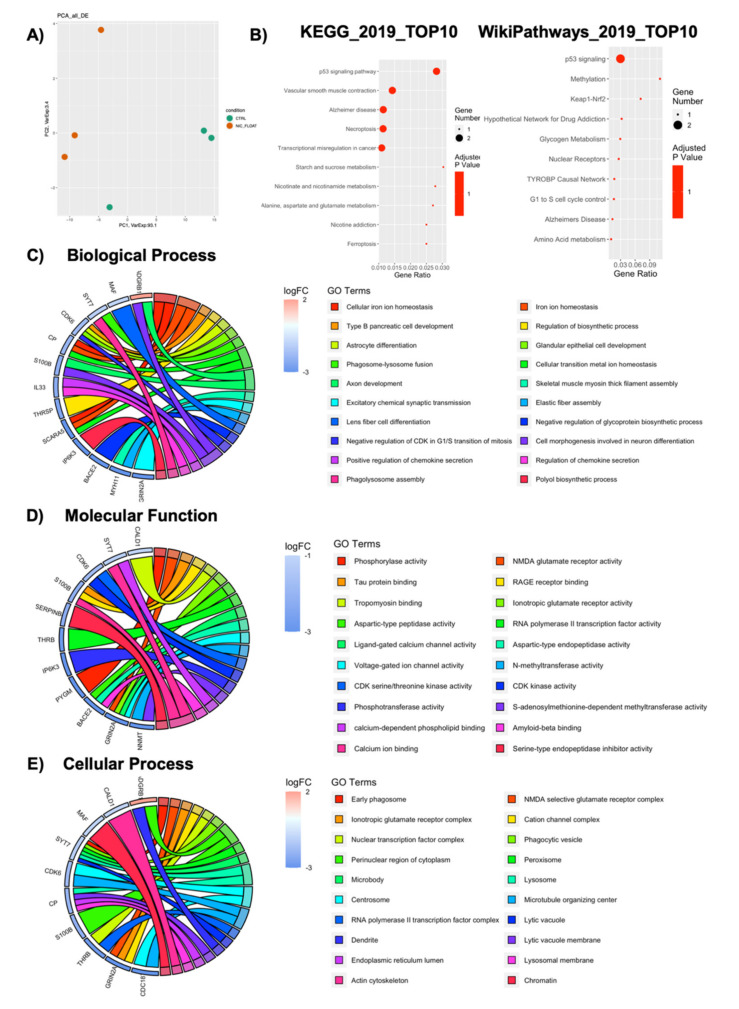
Computational analysis of neural precursor Cells detached from the Nichoid and brought back to standard floating conditions for 7 days. RNA-Seq was performed on three samples for condition (*n* = 3). Specifically, three experiments were performed each including one sample of NPCs grown in standard floating conditions for 7 days and one sample of NPCs grown inside the Nichoid and replated in standard floating condition for 7 more days. (**A**) Principal component analysis (PCA) of differently expressed genes in NPCs replated after Nichoid-growth and in standard conditions. We considered as differentially expressed only genes showing |log_2_(Nichoid samples/control samples)| ≥ 1 and a false discovery rate of ≤0.1. (**B**) Pathway analysis for DE genes in NPCs replated after Nichoid-growth to NPCs grown in standard conditions. The y-axis represents the name of the pathway, the x-axis represents the gene ratio, dot size represents the number of different genes, and the color indicates the adjusted *p*-value. Dot plot of top 10 deregulated pathways from KEGG and WikiPathways analysis. (**C**–**E**) GO analysis for DE genes in NPCs replated after Nichoid-growth compared to NPCs grown in standard conditions. (**C**) Top 20 enriched GO terms for Biological Process, (**D**) Molecular Function, and (**E**) Cellular Component are shown. The panels have been obtained considering all DE genes, and the color represents different pathways implicated to the deregulated genes.

As cells could potentially be detached and transplanted for clinical translation, we aimed to verify if scaffold-grown NPCs maintained a transcriptional deregulation even after cells were detached and brought back to standard floating conditions for 7 days. Thus, we performed a whole transcriptome analysis of NPCs replated after 7 days Nichoid-expansion. PCA analysis of the DE RNAs in NPCs replated after Nichoid-growth (Figure 9A) showed different expression profiles, suggesting that also in this condition, gene expression is deregulated. A total of 36 DE RNAs was identified, 34 of which were coding genes. Their functions are reported in Table 4. The two deregulated noncoding genes are Tpt1-ps3, a processed pseudogene with a log_2_FC = 1.59, and Gm16010, an antisense transcript with log_2_FC = −2.61. Both these genes’ functions are currently unknown.

The deregulated transcripts with a deregulation ≥1 in terms of |Log_2_FC| were subjected to pathways analysis via the Enrichr web tool [40]. We identified the top 10 deregulated pathways with the KEGG 2019 and WikiPathways 2019 tools (Figure 9B). Interestingly, we found some of the deregulated pathways reported in previous sections even after cells were detached and brought back to standard floating conditions for 7 days (e.g., p53 signaling pathway, methylation, nuclear receptors, Keap1-Nrf2, amino acid metabolism, and alanine, aspartate, and glutamate metabolism). When considering the top 20 GO terms, we identified 19 genes deregulated (13 for Biological Process, 12 for Molecular Function, and 10 for Cellular Component). With respect to the Biological Process, we identified 236 pathways (Table S8). Among the 20 GO terms, we found pathways involved in axon development and cell morphogenesis involved in neuron differentiation emerged as deregulated (Figure 9C). With respect to Molecular Function, we found 48 deregulated pathways (Table S9). Among them, the most enriched terms we found were “Phosphorylase activity,” “NMDA glutamate receptor activity,” and “Tau protein binding” (Figure 9D). With respect to Cellular Component, we found 25 deregulated pathways (Table S10). When considering the top 20 terms, the most enriched were “Early phagosome,” “NMDA selective glutamate receptor complex,” and “Ionotropic glutamate receptor complex.” Moreover, as for Cellular Component described in the previous sections, the analysis revealed a strong implication for the contractile cellular component, highlighting actomyosin, cytoskeleton, nuclear receptor complex, and chromatin. This demonstrates, again, that the maintenance inside the Nichoid strongly alters the cytoskeleton morphology, thus leading to an altered transcriptional cascade (Figure 9E).

## 3. Discussion

Three-dimensional scaffolds are becoming more and more relevant for their implications in regenerative medicine. In particular, the Nichoid scaffold, for its dimension and structure, is inspired by the natural stemness niche and aims to improve the therapeutic potential of stem cells [9,10]. We aimed to characterize the effect of the Nichoid on a specific kind of murine stem cells (namely, neural precursors cells, NPCs) well characterized and already tested for therapeutic efficacy in animal experimental models of neurodegenerative diseases [29,30,31,32]. We visualized the tight relations that these cells develop with the 3D niche, appreciating a change in morphology as the carpet-like structure that cells form inside the Nichoid is completely different from the typical spheroids organization in floating conditions. To gain more in-depth insights over the molecular basis for these alterations in cellular shape and interactions, we performed a full profiling of the deregulated transcripts via RNA-Seq. We first analyzed all the DE genes, and we found a total of 1934 DE RNAs, 81% (1577 out of 19,344) of which were coding genes. Amongst the noncoding genes, we found functional deregulated lincRNAs involved in pluripotency, cell survival, and gene expression (2900076A07Rik, Gm16892, Gm4262, Gm807, C130071C03Rik, Gm26917, Lncpint, and Linc-p21). Long noncoding RNAs could be crucial in regulating stem cell’s fate and neurodegenerative processes, as recent works are highlighting more and more a role for these molecules in these contexts [42,104,105,106]. Indeed, the Nichoid can affect cellular fate also regulating the noncoding genome, demonstrating to be able to cause a whole transcriptome alteration. 

Using the KEGG 2019, WikiPathways, and GO enrichment analysis, we were able to deeply investigate altered pathways, Molecular Functions, Biological Processes, and Cellular Components. We found that these analyses strongly indicated a role for the Nichoid in regulating mechanotransduction-associated signaling. The term mechanotransduction refers to the conversion of mechanic extracellular stimuli into an alteration in the cellular biology [107]. Going deeply through the analysis, we showed that the Nichoid’s effect starts at a membrane level but can also impact on signaling transduction and cellular metabolism, ending in an altered nuclear activity, basically leading to a global change in cellular organization and function. 

Indeed, many signaling pathways are activated in response to different forms of mechanical force being exerted on the cell surface [52]. Nichoid-grown NPCs present alterations in the signaling pathways related to this mechanical activation of the membrane, such as integrins, cadherins, focal adhesions, and even Rho activation, fundamental in promoting contractility and bundling of actin filaments [51]. These results indicate a stable interaction between cells and the scaffold that affects the organization of cytoskeleton. The deregulation of pathways related to cell–cell interaction (e.g., tight junction, Gap junction, and ECM-receptor interaction) indicates that the Nichoid influences not only the single cellular organization but also the organization and interaction between cells. Alteration in signaling pathways linked to membrane alterations (such as G-proteins and S1P signaling) can lead to activation of intracellular signaling transduction pathways, also correlated to mechanotransduction. Amongst these, relevant examples are given by the MAPK cascade, PI3K-Akt signaling pathway, AMPK signaling, Wnt signaling, and RAP1 signaling pathway, implicated in motility, proliferation, cell migration, cell polarity, and even integrin- and cadherin-regulated processes involved in mechanotransduction [59,60,61,62,63]. 

The deregulation in these abovementioned cell signaling pathways suggests an altered mechanical to biochemical signaling. Indeed, a really relevant alteration is observed in metabolic processes involving all classes of metabolites: lipids, carbohydrates, proteins, vitamins, and nucleotides biosynthesis. Interestingly, one work identified that the synthesis of neutral lipids is a general response to mechanical signals delivered by cell–matrix adhesions [67]. More specifically, the authors conclude that conditions of reduced actomyosin contractility lead to inhibition of Lipin-1, accumulation of SCAP/SREBP to the Golgi apparatus, and activation of SREBP transcription factors, thus driving lipid synthesis and accumulation. It is thus extremely interesting to note that Lpin1 (which codifies for Lipin-1) was found downregulated in the Nichoid-grown NPCs. The isotropic stimuli exerted on the cells, which in turn could lead to a condition of reduced actomyosin contractility, could possibly lead to a decrease in Lipin-1, responsible for the subsequent deregulation in these pathways involved in lipid biogenesis. The deregulation of carbohydrates and protein metabolism could be explained by a deregulation in the expression and thus activity of AMPK, which can be regulated by the activation of E-cadherin simply through mechanical forces [70]. Moreover, one work shows that physiological dynamic compressions in primary human osteoarthritic chondrocytes could influence amino acids metabolism [108]. Lastly, we show that the mechanically induced effect on cell biology also implicates the nucleus, as mechanotransduction can modulate the shape and the structure of the nucleus in response to changes in the interaction between cytoskeleton and nucleoskeleton [74]. We found multiple nuclear processes involved (DNA repair mechanisms, modulation of transcription, regulation of molecule transport through nuclear membrane, RNA metabolism, and activation of specific mechanosensitive gene), all already linked to mechanotransduction processes [78,79,80,81,82,83,84,85,86,87]. We assessed the differences in cytoskeleton organization and adhesion complexes formation evaluating the specific deregulation in genes’ pertaining to these processes, along with a morphological analysis with immunofluorescence and ESEM technology. We showed that FAK and Vinculin, key components of focal adhesion complexes, present a different distribution is n Nichoid-grown NPCs as opposed to those grown in standard floating conditions. We showed that NPCs expanded inside the Nichoid can be stimulated to differentiate into neuronal phenotype and can be detached, maintaining a transcriptional deregulation. These results allow us to speculate that NPCs could potentially be transplanted for clinical translation. 

The results shown refer to the first analysis of how the 3D scaffold Nichoid influences the biological and genetic response of stem cells. The Nichoid’s ability to induce changes in cultured stem cells at molecular level without any other chemical agents could be extremely important to characterize the essential role of the microenvironment and cell–cell interaction in the regulation of gene expression. Moreover, the Nichoid allows recreating the stem cells adhesion, migration, differentiation, proliferation, and cell signaling that best mimics physiological conditions. This capacity is essential for regenerative medicine based on development of stem cell therapies, thus highlighting the importance of the Nichoid studies.

## 4. Materials and Methods 

### 4.1. Nichoid Microfabrication

The Nichoid design is aimed at emulating the microenvironment of the native stem niche; Nichoids are fabricated so that cells are subjected to isotropic mechanical stimuli [11]. 

Nichoids were fabricated directly on circular glass coverslips of 12 mm in diameter with a direct laser writing technique known as two photon laser polymerization (2PP) as previously described by Zandrini et al. [12]. The use of 2PP technology allowed the creation of computer-designed 3D structure with a spatial resolution down to 100 nm.

Briefly, Nichoids were obtained by polymerizing a photoresist termed SZ2080, after its main elements silicon and zirconium and the molar ratio between the two components. Specifically, zirconium propoxide (Sigma-Aldrich, St. Louis, MO, USA) and methacryloxypropil trimethoxysilane (Sigma-Aldrich, St. Louis, MO, USA) were used to obtain a sol–gel-synthetized silicon–zirconium hybrid inorganic–organic resin. In particular, 1% concentration of Irg photoinitiator (Irgacure 369, 2-Benzyl-2-dimethylamino-1-(4-morpholinophenyl)-butanone-1) was added to enhance the photopolymerization process [109]. SZ2080 offers biocompatibility, good optical transmission, chemical and electrochemical inertia, and long-term stability [10,11,15]. The SZ2080 presents with a Young’s modulus around 0.14 GPa and is chemically inert [9,10,15].

The laser source employed during the Nichoid microfabrication was a laboratory-made Ytterbium (Yb) system, based on a cavity-dumped oscillator in mode-locking, Yb:KYW with a wavelength of 1030 nm. In Nichoid fabrication, the selected repetition rated was 1 MHz, the pulse power was 1 μJ, and the pulse duration was 300 fs [15].

Samples were fabricated directly on circular glass coverslips of 12 mm in diameter and the unpolymerized resin was removed placing them in a metallic cage and leaving them soaked for 20–25 min in a glass baker filled with 50% (v/v) methyl isobutyl ketone and 50% (*v*/*v*) isopropyl alcohol solution (Sigma-Aldrich, St. Louis, MO, USA) [15,110,111]. After that, samples were briefly and gently washed with isopropyl alcohol to remove any residues of unpolymerized resin and then dried with room temperature nitrogen and checked for quality with scanning electron microscope (SEM).

The use of 2PP allowed the creation of a 3D structure with a precise geometry. Specifically, the elementary unit of the Nichoid was a single niche, 30 μm high and 90 × 90 μm in transverse dimensions, and consisted of three layers of lattice lines aligned vertically with a uniform spacing of 15 μm and horizontally arranged to make up pores sized gradually from 10 and 20 to 30 μm (please see Figure S2). Moreover, 25 single niches (5 × 5 niches) make up a single Nichoid block. Nichoid blocks were fabricated to cover up 8 mm of the coverslips surface with a constant spacing of 15 μm. 

### 4.2. Substrate Preparation

The 2PP-patterned substrates (Nichoid) were washed thoroughly, kept for 20 min in deionized water, disinfected for 90 min in 70% ethanol (VWR, Radnor, PA, USA), washed repeatedly in sterile deionized water, and dried under UV for 90 min in sterile conditions. A total of 25 samples were fabricated for the following biological analysis and Table S11 reports their usage in each experiment.

### 4.3. Primary Cells Isolation and Culture

Neural precursors cells (NPCs) expressing green fluorescent protein (GFP) were isolated 6 h postmortem from adult C57BL/6-Tg(UBC-GFP)30Scha/J mice weighing 25–30 g (Charles River, Wilmington, MA, USA) as previously described [28,29,33,34]. All animals’ procedures conform to the European Communities Directive of September 2010 (2010/63/UE) and have been approved by the Review Committee of the University of Milan. The specific codes are: N° 778/2017 and 535/2017. NPCs were maintained in culture in Neurobasal Medium (GIBCO^TM^, Life Technologies, Carlsbad, CA, USA) containing 2% B-27 supplement (Life Technologies, Carlsbad, CA, USA), 2% L-glutamine (EuroClone, Pero, Italy), 1% penicillin and streptomycin (EuroClone, Pero, Italy), b-FGF (human recombinant, 20 ng/mL), and hEGF (human recombinant, 20 ng/mL). 

### 4.4. Cells’ Seeding in the Nichoid

To perform biological experiments and seed the cells inside the Nichoid, NPCs maintained in culture were harvested, collected by centrifugation (123× *g* for 10 min), mechanically dissociated, and counted with trypan blue reagent (Sigma-Aldrich, St. Louis, MO, USA). Specifically, 10,000 cells were seeded at the center of the Nichoid in a single drop of 35 μL NPCs medium. The multiwell was kept in the incubator for 1 h to allow cells to enter the 3D niches and then 465 μL of NPCs medium were added. The multiwells were kept in the incubator until the end of each specific biological experiment. 

### 4.5. Cells’ Detachment from the Nichoid and Proliferation Assay

Proliferation assay was performed by plating 10,000 NPCs either in standard floating condition or inside the Nichoid. Cells were counted and collected after 3, 7, 10, and 14 days in two experiments (N = 2). In order to collect NPCs, cells were washed with PBS (Life Technologies, Carlsbad, CA, USA) and 200 μL of citric saline solution 1× prepared from a stock of 10× solution of 1.35 M KCl (Fluka BioChemika, Buchs, Switzerland) and 0.15 M sodium citrate dihydrate (Sigma-Aldrich, St. Louis, MO, USA) were added for 10 min. Cells were then collected and pelleted (123× *g* for 10 min). Then, cells were counted using the trypan blue exclusion method at the four different time points. Specifically, cells resuspended in trypan blue were transferred in a Bürker chamber and examined by light microscopy and scored as able (live) or unable to exclude the dye (dead).

Moreover, to evaluate the different growth for both conditions, the cell doubling time was evaluated with the following formula:

$$d=\frac{ln\left(\frac{X_{f}}{X_{i}}\right)}{ln2}$$

where $X_{f}$ and $X_{i}$ were, respectively, the final and the initial number of cells for each condition.

### 4.6. Environmental Scanning Electron Microscopy

For the environmental scanning electron microscopy (ESEM), two Nichoids samples were used and 6 fields were acquired for each Nichoid (*n* = 12). ESEM analysis was performed by plating 10,000 NPCs either in inside the Nichoid and keeping the samples in NPCs medium for 7 days. Samples were fixed by dehydration with ethanol. More specifically, the culture medium was removed and the samples were incubated for 2 h at room temperature with a solution composed of 1.5% (*v*/*v*) glutaraldehyde 50% (*v*/*v*) and 0.1 M sodium cacodylate (pH = 7.1–7.2). The samples were rinsed with 0.1 M sodium cacodylate buffer. The samples were rinsed with 0.1 M sodium cacodylate buffer and incubated for 5 min in increasing ethanol concentrations (20–30–40–50–60–70–80–90–96–100% *v*/*v*). This passage was repeated twice. The images were acquired using the ESEM ZEISS EVO 50 EP. 

### 4.7. RNA Extraction

Total RNA from cultured cells was isolated using TRIzol Reagent (Invitrogen, Carlsbad, CA, USA) following standard protocol. RNA quality was assessed using a spectrophotometer (NANOPhotometer^®^ NP80, IMPLEN, Westlake Village, CA, USA) and a 2100 Bioanalyzer (Agilent RNA 6000 Nano Kit, Waldbronn, Germany); RNAs with a 260:280 ratio of ≥1.5 and an RNA integrity number of ≥8 were subjected to deep sequencing.

### 4.8. Libraries Preparation for RNA-Seq and Bioinformatic Data Analysis

For RNA-Seq, three samples for condition were analyzed (*n* = 3). Specifically, three experiments were performed each including one sample of NPCs grown in standard floating conditions for 7 days, one sample of NPCs grown inside the Nichoid for 7 days, and one sample of NPCs grown inside the Nichoid and replated in standard floating condition for 7 more days. Sequencing libraries were prepared with TruSeq Stranded Total RNA kit (Illumina, San Diego, CA, USA) using 200 ng total RNA. Qualities of sequencing libraries were assessed with 4200 TapeStation with the DNA1000 reagent kit. RNA processing was carried out using Illumina NextSeq 500 Sequencing. FASTQ files were generated via Illumina bcl2fastq2 (Version 2.17.1.14-https://support.illumina.com/downloads/bcl2fastq-conversion-software-v2–20.html, San Diego, CA, USA) starting from raw sequencing reads produced by Illumina NextSeq sequencer. Quality of individual sequences were evaluated using FastQC software (see Code Availability 1) after adapter trimming with Cutadapt software. Gene and transcript intensities were computed using STAR/RSEM software [112] using GENCODE Release M24 (GRCm38) as a reference, using the “-strandness forward” option. Differential expression analysis for mRNA was performed using R package DESeq2 [113], selected because of its superior performance in identifying isoforms differential expression [114]. Genes were considered differentially expressed and retained for further analysis with |Log_2_(Nichoid sample/control sample)| ≥ 1 and a FDR ≤ 0.1. We imposed minimum |Log_2_FC| of 1 and an FDR lower than 0.1 as thresholds to differentially expressed genes. This choice is motivated by the decision to maximize the sensitivity of this analysis, in order to perform a massive screening and identify candidate genes to be validated with a wider sample population with real-time analysis. The raw data obtained from the RNA-Seq analysis are deposited on the Gene Expression Omnibus repository (Appendix A).

### 4.9. Pathway and Coexpression Analysis

We performed a KEGG pathway analysis (Kyoto Encyclopedia of Genes and Genomes http://www.genome.ad.jp/kegg) and a WikiPathways analysis of differentially expressed coding genes via Enrichr web tool (https://maayanlab.cloud/Enrichr/, 28th July 2020) [43,115].

### 4.10. Real-Time PCR

For RNA-Seq validation with real-time PCR, eight samples for condition were analyzed (*n* = 8). Specifically, four experiments were performed each including two samples of NPCs grown in standard floating conditions for 7 days, two samples of NPCs grown inside the Nichoid for 7 days, and two samples of NPCs grown inside the Nichoid and replated in standard floating condition for 7 more days. Total RNA (500 ng) was reverse transcribed using iScript cDNA Synthesis Kit (Bio-Rad, Hercules, CA, USA) according to the manufacturer’s instructions. Using gene sequences available from NCBI for target genes (http://www.ncbi.nlm.nih.gov/nucleotide, 29 May 2020) PCR oligonucleotide primers for target genes were selected and primers are reported in Table S12. This was done with the NCBI’s Primer-BLAST tool. Real-time PCR was performed with StepOnePlus^TM^ Real-Time PCR System (Thermo Fisher, Waltham, MA, USA) using SSOSYBR Green Supermix (Bio-Rad, Hercules, CA, USA). Genes were quantified in triplicates, GAPDH was used as housekeeping gene for mouse samples. Gene expression was calculated using the 2^−ΔΔCt^ method.

### 4.11. Primary Cells Isolation and Differentiation

NPCs obtained from 2-months-old CD-1 mice (Charles River, Calco, Italy) were maintained in culture in the previously mentioned medium. Differentiation of NSCs was performed by plating the dissociated stem cells, at the density of 15,000 cells/cm^2^ in presence of adhesion molecules (Matrigel™, BD Biosciences, San Jose, CA, USA), and bFGF (10 ng/mL) for 48 h, and then, cells were exposed to the same medium without bFGF and with the addition of fetal bovine serum (2% vol/vol; EuroClone, Pero, Italy) for the following 5 days as previously described [28]. 

### 4.12. Immunocytochemistry Analysis for Differentiated Cells

Cells differentiated inside the Nichoid for 7 days as described previously [28], then medium was removed and cells were fixed with 4% paraformaldehyde (BDH, Poole, UK) in 0.1 M PBS, pH 7.4 (Life Technologies, Carlsbad, CA, USA), for 10 min at room temperature, then were fixed for other 10 min in 2% paraformaldehyde and washed with PBS. Samples were incubated overnight at 4 °C in PBS containing 10% normal goat serum (NGS, Thermo Fisher, Waltham, MA, USA), 0.3% Triton X-100 (BDH, Poole, UK), and the appropriate primary antibody. Cells’ characteristics were assessed by immunocytochemistry with antibodies against Nestin (monocl. 1:200; Millipore MAB353-2444141, Burlington, MA, USA), microtubule-associated protein 2 (MAP2, polycl. 1:1000; Millipore Ab5622, Burlington, MA, USA). After thorough washing with PBS and 10% NGS, cells were incubated for 90 min at room temperature with the appropriate secondary antibody (Alexa Fluor 488 and 546, Life Technologies, Carlsbad, CA, USA). Nuclei were stained with 4′,6-diamidino-2-phenylindole (DAPI 1 µg/mL) (Sigma-Aldrich, St. Louis, MO, USA), 10 min at room temperature, mounted using the FluorSave Reagent (Calbiochem, Merck Chemical, Darmstadt, Germany), and analyzed by confocal microscopy (confocal laser scanning microscope Olympus FluoView FV10i, Segrate, Italy). The ImageJ software (Version 2.1.0/1.53c, NIH, Bethesda, MD, USA) was used for microphotographic digital analysis. In control determinations, primary antibody was omitted and replaced with equivalent concentrations of unrelated IgG of the same subclass. The positive pixels were quantified against the negative background. The microscope light intensity of the laser was the same for all analyzed sections and for determining the background optical density. Immunocytochemistry analysis was performed acquiring three different fields in each sample, one sample per marker (*n* = 3).

### 4.13. Immunocytochemistry Analysis for NPCs in Standard Conditions 

For NPCs grown in standard floating conditions, 1 mL of culture medium containing spheroids was fixed with 108 µL of 37% paraformaldehyde (BDH, Poole, UK) for 15 min at room temperature. Spheroids were then centrifuged for 5 min at 6000–10,000 rpm, and the supernatant was discarded. Samples were permeabilized in a solution containing 300 mM sucrose, 0.2% Triton X-100, and PBS for 15 min and then centrifuged for 5 min at 6000–10,000 rpm. The supernatant was discarded and the pellet was blocked in 2% BSA diluted in PBS for 15 min at room temperature. Again, samples were centrifuged for 5 min at 6000–10,000 rpm, the supernatant was discarded, and the pellet was incubated with binding buffer (0.2% BSA diluted in PBS) with an appropriate primary antibody. Specifically, the following antibodies were used: Nestin (monocl. 1:200; Millipore MAB353-2444141, Burlington, MA, USA), FAK (polycl. 1:100; Santa Cruz sc-932, Santa Cruz, CA, USA), and Vinculin (monocl. 1:150; Abcam ab129002, Cambridge, UK). Samples were incubated overnight at 4 °C on a rocking platform and then centrifuged for 5 min at 2000–4000 rpm. The pellet was washed in binding buffer for 5 min at 4000–7000 rpm, the supernatant was discarded, and binding buffer was added using the appropriate secondary antibody (Alexa Fluor 546, Life Technologies, Carlsbad, CA, USA). Samples were placed in agitation for 90 min and centrifuged for 5 min at 4000–7000 rpm; then, the supernatant was discarded and pellet was washed (centrifuged at 4000–6000 rpm) twice with binding buffer. The supernatant was discarded and samples were resuspended in a solution containing 90% glycerol, 5% PBS, and 5% DAPI (DNA-binding dye 4′-6′-diamidino-2-phenylindole). Samples were then mounted on a microscope slide and analyzed by confocal microscopy (confocal laser scanning microscope Olympus FluoView FV10i, Segrate, Italy). For NPCs plated inside the Nichoids, the protocol was the same as in Section 4.12, using the following primary antibodies: Nestin (monocl. 1:200; Millipore MAB353-2444141, Burlington, MA, USA), FAK (polycl. 1:100; Santa Cruz sc-932, Santa Cruz, CA, USA), and Vinculin (monocl. 1:150; Abcam ab129002, Cambridge, UK). The ImageJ software was used for microphotographic digital analysis and surface plot analysis. In control determinations, primary antibody was omitted and replaced with equivalent concentrations of unrelated IgG of the same subclass. The positive pixels were quantified against the negative background. The microscope light intensity of the laser was the same for all analyzed sections and for determining the background optical density.

### 4.14. Statistical Analysis

Data were expressed as mean ± SEM. The statistical analysis was performed with Student’s *t*-test. The Prism 7 software (GraphPad Software Inc., La Jolla, CA, USA) was used assuming a *p*-value less than 0.05 as the limit of significance.

## 5. Conclusions

The results hereby reported highlight the Nichoid’s ability to induce changes in cultured stem cells at any molecular level without the need of chemical additive of xenogeneic origin, allowing recreating the physiological microenvironment where stem cells reside. These results could highly impact the broader field of regenerative medicine, thus highlighting the importance of Nichoid studies in stem cells-based therapies.

## Figures and Tables

**Table 1:** Number of deregulated coding and noncoding RNAs after transcriptome analysis.

	CTR vs. NIC	
	mRNAs	ncRNAs
**Upregulated**	927	147
**Downregulated**	650	210
**Total**	1577	357

**Table 2:** Gene description and fold change of top 10 deregulated RNAs after transcriptome analysis.

Gene Name	log_2_FC	Gene Description
Clcf1	7.45	Protein that in complexation with CRLF1 forms a heterodimeric neurotropic cytokine that plays a crucial role during neuronal development. Stimulates B-cells. Binds to and activates the ILST/gp130 receptor.
Ceacam1	−7.25	Cell adhesion protein that mediates homophilic cell adhesion in a calcium-independent manner. Plays a role as coinhibitory receptor in immune response and insulin action, and is an activator of angiogenesis. Downregulates cell growth in response to EGF through its interaction with SHC1. Inhibits cell migration and cell scattering through interaction with FLNA, interfering with FLNA’s interaction with RALA.
St18	7.21	Repressor that binds to DNA sequences containing a bipartite element consisting of a direct repeat of the sequence 5′-AAAGTTT-3′ separated by 2–9 nucleotides.
Slc38a1	−7.19	Functions as a sodium-dependent amino acid transporter. May supply glutamatergic and GABAergic neurons with glutamine, which is required for the synthesis of the neurotransmitters, e.g., glutamate and GABA
Sema3e	7.05	Plays an important role in signaling via the cell surface receptor PLXND1. Mediates reorganization of the actin cytoskeleton, leading to the retraction of cell projections. Promotes focal adhesion disassembly and inhibits adhesion of endothelial cells to the extracellular matrix.
Actl7b	6.35	Actin-like protein 7B, belongs to the actin family
Scrt2	6.14	Transcriptional repressor scratch 2; plays a role in DNA binding and negative regulation of transcription
Aldh1a3	5.69	NAD-dependent aldehyde dehydrogenase that catalyzes the formation of retinoic acid
Fgl1	−5.64	Immune suppressive molecule that inhibits antigen-specific T-cell activation by acting as a major ligand of LAG3
Lhx6	5.63	Probable transcription factor required for the expression of a subset of genes involved in interneurons migration and development. Functions in the specification of cortical interneuron subtypes and in the migration of GABAergic interneuron precursors from the subpallium to the cerebral cortex

Protein function description was obtained from the UniProtKB database (https://www.uniprot.org/uniprot).

**Table 3:** Number and biotype of deregulated noncoding RNAs.

	ncRNAs		
	Upregulated	Downregulated	Total
Antisense	11	16	27
Bidirectional promoter lncRNA	0	3	3
lincRNA	20	40	60
miRNA	2	1	3
miscRNA	1	0	1
Processed pseudogene	11	14	25
Processed transcript	17	28	45
rRNA	1	0	1
Sense intronic	9	3	12
snoRNA	1	31	32
snRNA	3	6	9
TEC	67	51	118
Transcribed unprocessed pseudogene	3	6	9
Unitary pseudogene	0	3	3
Unprocessed pseudogene	1	8	9
	147	210	357

**Table 4:** Gene description and fold change of deregulated RNAs after transcriptome analysis.

Gene Name	log_2_FC	Gene Description
**Megf6**	1.88	Multiple epidermal growth factor-like domains protein 6; multiple EGF-like-domains 6
**Adgrb1**	1.62	Cell adhesion protein that mediates homophilic cell adhesion in a calcium-independent manner. Plays a role as coinhibitory receptor in immune response and insulin action and is an activator of angiogenesis. Downregulates cell growth in response to EGF through its interaction with SHC1. Inhibits cell migration and cell scattering through interaction with FLNA, interfering with FLNA’s interaction with RALA
**Unc80**	1.45	Protein unc-80 homolog; component of the NALCN sodium channel complex, required for channel regulation. This complex is a cation channel activated by neuropeptides substance P, neurotensin, and extracellular calcium that regulates neuronal excitability by controlling the sizes of NALCN-dependent sodium-leak current. UNC80 is essential for NALCN sensitivity to extracellular calcium; Belongs to the unc-80 family
**Cad**	1.05	CAD protein; this protein is a “fusion” protein encoding four enzymatic activities of the pyrimidine pathway (GATase, CPSase, ATCase, and DHOase)
**Cald1**	−1.11	Caldesmon 1
**Maf**	−1.13	Transcription factor Maf; acts as a transcriptional activator or repressor. When overexpressed, represses antioxidant response element (ARE)-mediated transcription. Involved either as an oncogene or as a tumor suppressor, depending on the cell context. Involved in embryonic lens fiber cell development. Recruits the transcriptional coactivators CREBBP and/or EP300 to crystallin promoters leading to upregulation of crystallin gene during lens fiber cell differentiation
**Syt7**	−1.28	Synaptotagmin-7; Ca(2+) sensor involved in Ca(2+)-dependent exocytosis of secretory and synaptic vesicles through Ca(2+) and phospholipid binding to the C2 domain. Ca(2+) induces binding of the C2 domains to phospholipid membranes and to assembled SNARE-complexes; both actions contribute to triggering exocytosis. SYT7 binds Ca(2+) with high affinity and slow kinetics compared to other synaptotagmins
**Ifi35**	−1.47	Interferon-induced protein 35
**Ccser1**	−1.70	Serine-rich coiled-coil domain-containing protein 1; coiled-coil serine rich 1
**Txlnb**	−1.79	Beta-taxilin; promotes motor nerve regeneration. May be involved in intracellular vesicle traffic
**Cdk6**	−1.82	Cyclin-dependent kinase 6; serine/threonine-protein kinase involved in the control of the cell cycle and differentiation; promotes G1/S transition. Phosphorylates pRB/RB1 and NPM1. Interacts with D-type G1 cyclins during interphase at G1 to form a pRB/RB1 kinase and controls the entrance into the cell cycle. Involved in initiation and maintenance of cell cycle exit during cell differentiation; prevents cell proliferation and regulates negatively cell differentiation, but is required for the proliferation of specific cell types (e.g., erythroid and hematopoietic cells)
**Gbp9**	−1.95	Guanylate-binding protein 9
**Cp**	−2.00	Ceruloplasmin; ceruloplasmin is a blue, copper-binding (6–7 atoms per molecule) glycoprotein. It has ferroxidase activity oxidizing Fe(2+) to Fe(3+) without releasing radical oxygen species. It is involved in iron transport across the cell membrane. Provides Cu(2+) ions for the ascorbate-mediated deaminase degradation of the heparan sulfate chains of GPC1. May also play a role in fetal lung development or pulmonary antioxidant defense
**S100b**	−2.16	Protein S100-B; weakly binds calcium but binds zinc very tightly—distinct binding sites with different affinities exist for both ions on each monomer. Physiological concentrations of potassium ion antagonize the binding of both divalent cations, especially affecting high-affinity calcium-binding sites. Binds to and initiates the activation of STK38 by releasing autoinhibitory intramolecular interactions within the kinase. Interaction with AGER after myocardial infarction may play a role in myocyte apoptosis by activating ERK1/2 and p53/TP53 signaling
**Serpinb8**	−2.25	Serpin B8; has an important role in epithelial desmosome-mediated cell–cell adhesion
**Il33**	−2.29	Interleukin-33; cytokine that binds to and signals through the IL1RL1/ST2 receptor, which in turn activates NF- κ -B and MAPK signaling pathways in target cells. Involved in the maturation of Th2 cells inducing the secretion of T-helper type 2-associated cytokines. It is also involved in activation of mast cells, basophils, eosinophils, and natural killer cells. Acts as a chemoattractant for Th2 cells, and may function as an “alarmin,” which amplifies immune responses during tissue injury
**Thrsp**	−2.50	Thyroid hormone-inducible hepatic protein; plays a role in the regulation of lipogenesis, especially in lactating mammary gland. Important for the biosynthesis of triglycerides with medium-length fatty acid chains. May modulate lipogenesis by interacting with MID1IP1 and preventing its interaction with ACACA. May function as transcriptional coactivator
**Cmbl**	−2.53	Carboxymethylenebutenolidase homolog; cysteine hydrolase
**Nupr1**	−2.60	Nuclear protein 1; chromatin-binding protein that converts stress signals into a program of gene expression that empowers cells with resistance to the stress induced by a change in their microenvironment. Interacts with MSL1 and inhibits its activity on histone H4 ‘Lys-16′ acetylation (H4K16ac). Binds the RELB promoter and activates its transcription, leading to the transactivation of IER3.
**Thrb**	−2.81	Thyroid hormone receptor beta; nuclear hormone receptor that can act as a repressor or activator of transcription. High-affinity receptor for thyroid hormones, including triiodothyronine and thyroxine
**Olfr287**	−3.00	Olfactory receptor 287
**Srl**	−3.16	Sarcalumenin; may be involved in the regulation of calcium transport
**Scara5**	−3.27	Scavenger receptor class A member 5; ferritin receptor that mediates nontransferrin-dependent delivery of iron. Mediates cellular uptake of ferritin-bound iron by stimulating ferritin endocytosis from the cell surface with consequent iron delivery within the cell. Delivery of iron to cells by ferritin is required for the development of specific cell types, suggesting the existence of cell type-specific mechanisms of iron traffic in organogenesis, which alternatively utilize transferrin or nontransferrin iron delivery pathways
**Ip6k3**	−3.33	Inositol hexakisphosphate kinase 3; converts inositol hexakisphosphate (InsP6) to diphosphoinositol pentakisphosphate (InsP7/PP-InsP5). Converts 1,3,4,5,6-pentakisphosphate (InsP5) to PP-InsP4 (by similarity)
**Pygm**	−3.45	Glycogen phosphorylase, muscle form; phosphorylase is an important allosteric enzyme in carbohydrate metabolism
**Otoa**	−3.55	Otoancorin; may act as an adhesion molecule; belongs to the stereocilin family
**Bace2**	−3.72	Beta-secretase 2; responsible for the proteolytic processing of the amyloid precursor protein (APP). Cleaves APP, between residues 690 and 691, leading to the generation and extracellular release of beta-cleaved soluble APP, and a corresponding cell-associated C-terminal fragment, which is later released by gamma-secretase
**Myh11**	−3.78	Myosin-11; muscle contraction; belongs to the TRAFAC class myosin-kinesin ATPase superfamily. Myosin family
**Grin2a**	−4.45	Glutamate receptor ionotropic, NMDA 2A; component of NMDA receptor complexes that function as heterotetrameric, ligand-gated ion channels with high calcium permeability and voltage-dependent sensitivity to magnesium. Channel activation requires binding of the neurotransmitter glutamate to the epsilon subunit, glycine binding to the zeta subunit, plus membrane depolarization to eliminate channel inhibition by Mg(2+)
**Ccdc187**	−4.53	Coiled-coil domain-containing protein 187; RIKEN cDNA 4932418E24 gene
**Wif1**	−5.10	Wnt inhibitory factor 1; binds to WNT proteins and inhibits their activities. May be involved in mesoderm segmentation
**Nnmt**	−5.15	Nicotinamide N-methyltransferase; catalyzes the N-methylation of nicotinamide and other pyridines to form pyridinium ions. This activity is important for biotransformation of many drugs and xenobiotic compounds; belongs to the class I-like SAM-binding methyltransferase superfamily. NNMT/PNMT/TEMT family
**Gm14406**	−6.56	Predicted gene 14406
**Defb41**	−8.64	Beta-defensin 41; has bactericidal activity; belongs to the beta-defensin family

Protein function description was obtained from the UniProtKB database (https://www.uniprot.org/uniprot).

## Supplementary Materials

Supplementary materials can be found at https://www.mdpi.com/1422-0067/21/18/6775/s1.

## Abbreviations

3D	Three-dimensional
AMPK	AMP-activated protein kinase
ceRNA	Competing endogenous RNA
DE RNAs	Differentially expressed coding and noncoding RNAs
ECM	Extracellular matrix
ESEM	Environmental scanning electron microscopy
GO	Gene Ontology
GPCRs	G-protein coupled receptors
KEGG	Kyoto Encyclopedia of Genes and Genome
lncRNA	Long noncoding RNA
NPCs	Neural precursors stem cells
S1P	Sphingosine-1-phosphate
TGF-β	Transforming growth factor-beta

## Appendix A

The raw data obtained from the RNA-Seq analysis are deposited on the Gene Expression Omnibus repository (GSE150767, GSE157897).
