# Supplementary material for: Dissecting the Effect of a 3D Microscaffold on the Transcriptome of Neural Stem Cells with Computational Approaches: A Focus on Mechanotransduction
Source: Int J Mol Sci. 2020 Sep 15;21(18):6775. doi: 10.3390/ijms21186775 (PMC7555048; doi:10.3390/ijms21186775)
Supplement: Supplementary file 1 [file ijms-21-06775-s001.zip › Rey et al suppl files/SupplementaryTable11-Number of Nichoids.docx]

| Biological applications | Number of Nichoids |
| --- | --- |
| Proliferation assays | 8 |
| RNA-Sequencing | 3 |
| ESEM | 2 |
| Real Time PCR | 8 |
| Immunofluorescence analysis | 4 |
