# Supplementary material for: Dissecting the Effect of a 3D Microscaffold on the Transcriptome of Neural Stem Cells with Computational Approaches: A Focus on Mechanotransduction
Source: Int J Mol Sci. 2020 Sep 15;21(18):6775. doi: 10.3390/ijms21186775 (PMC7555048; doi:10.3390/ijms21186775)
Supplement: Supplementary file 1 [file ijms-21-06775-s001.zip › Rey et al suppl files/Supplementary Table 12.docx]

| Gapdh-FW | CCAGGGCTGCCATTTGCAGTGGCAAAGTGG |
| --- | --- |
| Gapdh-REV | CCTGGAAGATGGTGATGGGCTTCCCGTTGA |
| Cntn2-FW | GAGCGCATCCTGATGCAGAG |
| Cntn2-REV | AGACACACCTAGAGTGCGGG |
| Pard6b-FW | GGTGAAGAGCAAGTTTGGAGC |
| Pard6b-REV | ACATGTTGCAGCAGTCCGTA |
| Il6ra-FW | CTTGGATAGAGCCCAGGACCAC |
| ll6ra-REV | CTGATCCTCGTGGTTGGCAG |
| Itga6-FW | CTGGAAAGGGATCGTCCGTG |
| Itga6-REV | AACGGGCACGAGACTTTCAT |
| Rarb-FW | CCCTCCTGGATTAACAGCC |
| Rarb-REV | TCCTGTCCAAAGCAAGCACA |
