# Supplementary figures and images for: Dissecting the Effect of a 3D Microscaffold on the Transcriptome of Neural Stem Cells with Computational Approaches: A Focus on Mechanotransduction
Source: Int J Mol Sci. 2020 Sep 15;21(18):6775. doi: 10.3390/ijms21186775 (PMC7555048; doi:10.3390/ijms21186775)

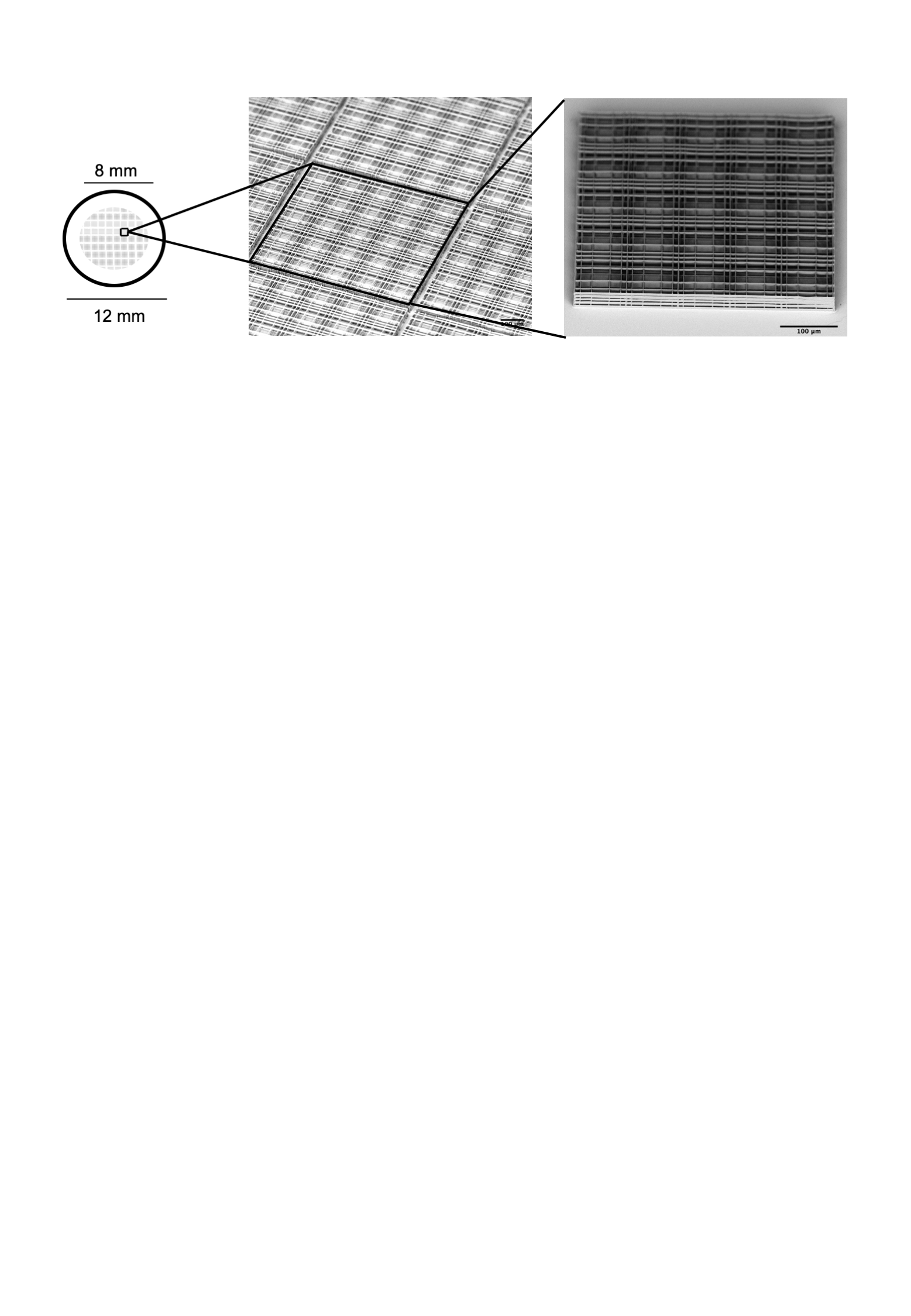

Supplement: Supplementary file 1 [file ijms-21-06775-s001.zip › Rey et al suppl files/Supplementary Figure 2.tiff]

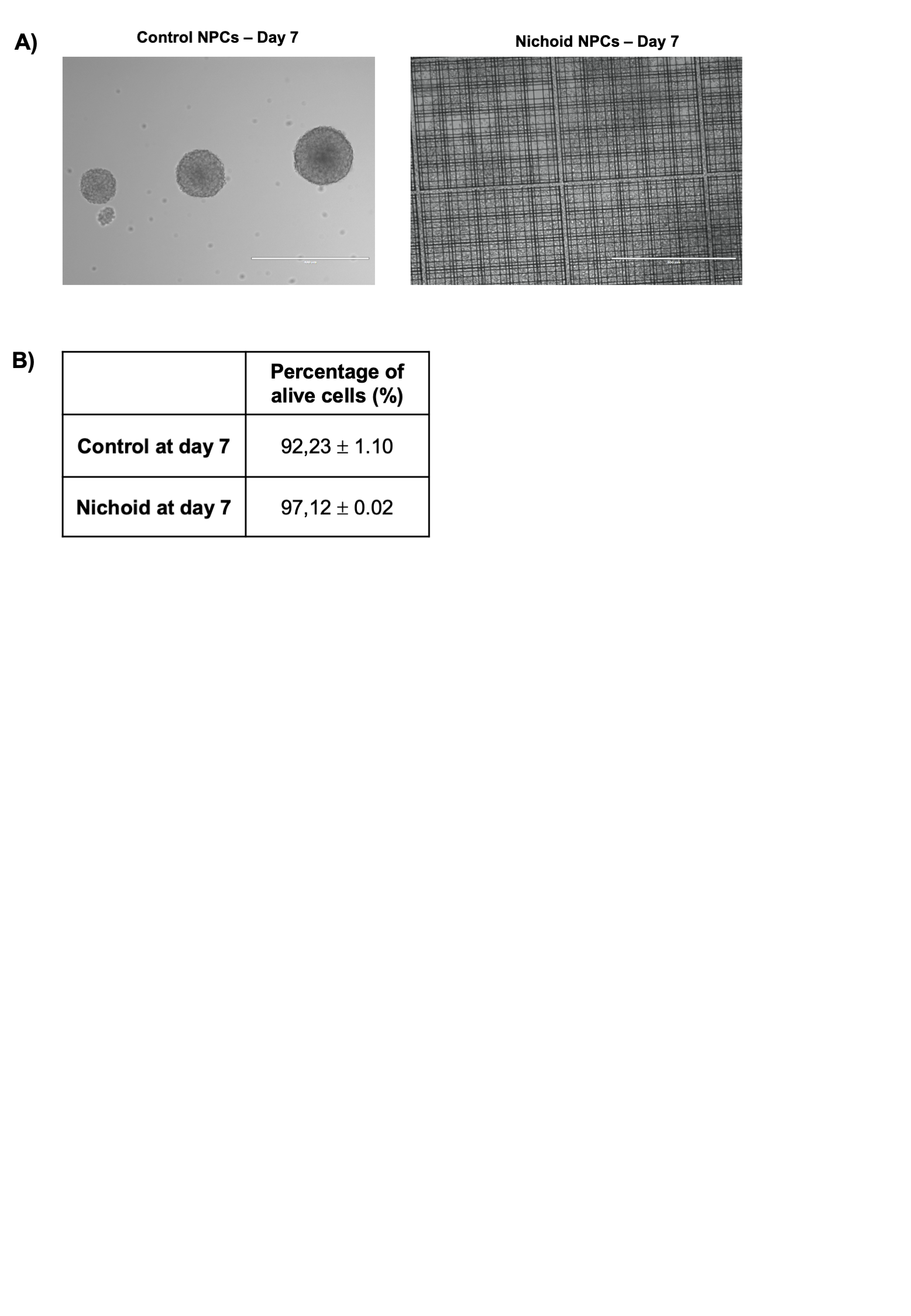

Supplement: Supplementary file 1 [file ijms-21-06775-s001.zip › Rey et al suppl files/Supplementary Figure 1.tiff]
